# 17β-estradiol in colorectal cancer: friend or foe?

**DOI:** 10.1186/s12964-024-01745-0

**Published:** 2024-07-19

**Authors:** Zihong Wu, Chong Xiao, Jiamei Wang, Min Zhou, Fengming You, Xueke Li

**Affiliations:** 1 Hospital of Chengdu University of Traditional Chinese Medicine, Chengdu, 610072 China; 2 TCM Regulating Metabolic Diseases Key Laboratory of Sichuan Province, Hospital of Chengdu University of Traditional Chinese Medicine, Chengdu, 610072 China; 3 Department of Obstetrics and Gynecology, Women and Children’s Hospital of Chongqing Medical University (Chongqing Health Center for Women and Children), Chongqing, 401147 China; 4 Oncology Teaching and Research Department of Chengdu, University of Traditional Chinese Medicine, Chengdu, 610072 China

**Keywords:** Colorectal cancer, 17β-estradiol, Dual roles, Cellular processes, Tumor microenvironment

## Abstract

Colorectal cancer (CRC) is a common gastrointestinal malignancy with higher incidence and mortality rates in men compared to women, potentially due to the effects of estrogen signaling. There is substantial evidence supporting the significant role of 17β-Estradiol (E2) in reducing CRC risk in females, although this perspective remains debated. E2 has been demonstrated to inhibit CRC cell proliferation and migration at the cellular level by enhancing DNA mismatch repair, modulating key gene expression, triggering cell cycle arrest, and reducing activity of migration factors. Furthermore, E2 contributes to promote a tumor microenvironment unfavorable for CRC growth by stimulating ERβ expression, reducing inflammatory responses, reversing immunosuppression, and altering the gut microbiome composition. Conversely, under conditions of high oxidative stress, hypoxia, and nutritional deficiencies, E2 may facilitate CRC development through GPER-mediated non-genomic signaling. E2’s influence on CRC involves the genomic and non-genomic signals mediated by ERβ and GPER, respectively, leading to its dual roles in anticancer activity and carcinogenesis. This review aims to summarize the potential mechanisms by which E2 directly or indirectly impacts CRC development, providing insights into the phenomenon of sexual dimorphism in CRC and suggesting potential strategies for prevention and treatment.

**Graphical Abstract:**

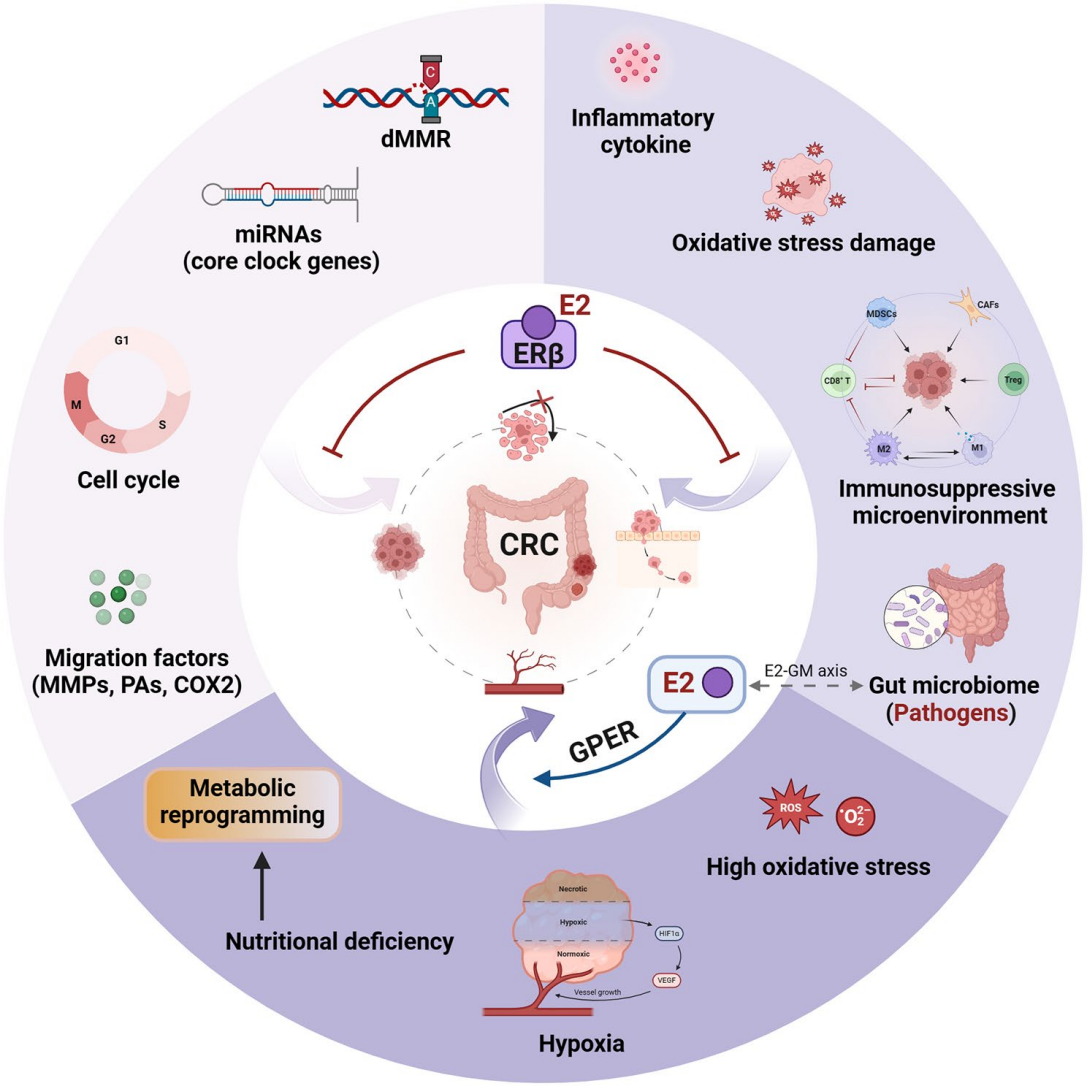

## Introduction

Colorectal cancer (CRC) represents a significant public health challenge worldwide, with high rates of morbidity and mortality [1]. Epidemiological studies have shown significant sex differences in CRC incidence and outcomes [2, 3]. From 2015 to 2019, men have shown approximately 30–40% higher annual incidence and mortality rates compared to women, along with slightly lower five-year survival rates [1, 3]. Individuals who have taken oral contraceptives or undergone postmenopausal hormone replacement therapy (HRT) have exhibited a 20-40% lower risk of CRC compared to those who have not undergone these treatments [4]. Moreover, postmenopausal patients tend to have lower levels of serum free estradiol and total 17β-estradiol (E2), which correlates with later tumor stagese [4–6]. These clinical findings underscore the protective effect of estrogen, particularly E2, in reducing CRC risk in women. However, the role of E2 in CRC remains controversial. Some studies have found no significant association between E2 levels and CRC risk [7–9], while others suggest that E2 might contribute to malignant progression and poorer outcomes in CRC [10, 11]. Despite most evidence supporting the link between HRT and reduced CRC risk in women, conflicting reports exist, indicating that the protective effect may vary among specific molecular subgroups [12, 13]. The intricate relationship between CRC and estrogen, coupled with limited and conflicting data on the association between endogenous estrogen and CRC incidence, underscores the need for a more thorough and scientific understanding of estrogen’s pathobiological role in CRC.

E2 is a principal estrogen in females, playing various physiological roles. Its molecular structure consists of an 18-carbon steroidal framework with the 17th hydroxyl group in the β-configuration, contributing to its important biological functions [14]. The synthesis and metabolism of E2 involve a complex series of enzymatic reactions [15]. Specifically, steroid sulfatase (STS) converts estrone sulfate (E_1_S) into estrone (E_1_), which is then reduced to E2 by the action of 17β-hydroxysteroid dehydrogenases (HSD17B1, HSD17B7, and HSD17B12) [14]. Conversely, E2 can be metabolized back to E1 *via* HSD17B2. As the most potent estrogen, E2 mediates its effects by binding to nuclear estrogen receptors (ERα, ERβ) and the membrane-associated G protein-coupled estrogen receptor (GPER). Recent studies have shown that intestinal epithelial cells possess the capability to metabolize estrogen, and fluctuations in E2 levels are closely associated with the onset and progression of CRC. In this review, we first outline the structures of the three principal ERs. Then, we discuss the detailed molecular and cellular mechanisms through which E2 promotes DNA mismatch repair, influences epigenetic modifications, triggers cell cycle arrest, initiates apoptosis, and suppresses cell proliferation and migration. Furthermore, we review the impact of E2 on the colorectal tumor microenvironment (TME), focusing on its effects on inflammation, immunity, and the gut microbiome. Lastly, we discuss the non-genomic effects of GPER-mediated E2 signaling on CRC. Overall, this review aims to explore both the direct and indirect, as well as genomic and non-genomic, actions of E2 in CRC development to offer novel insights into the sex differences in CRC risk and outline implications for prevention and treatment strategies.

## Estrogen receptors: ERα, ERβ and GPER

### ERα, ERβ

**Fig. 1:**
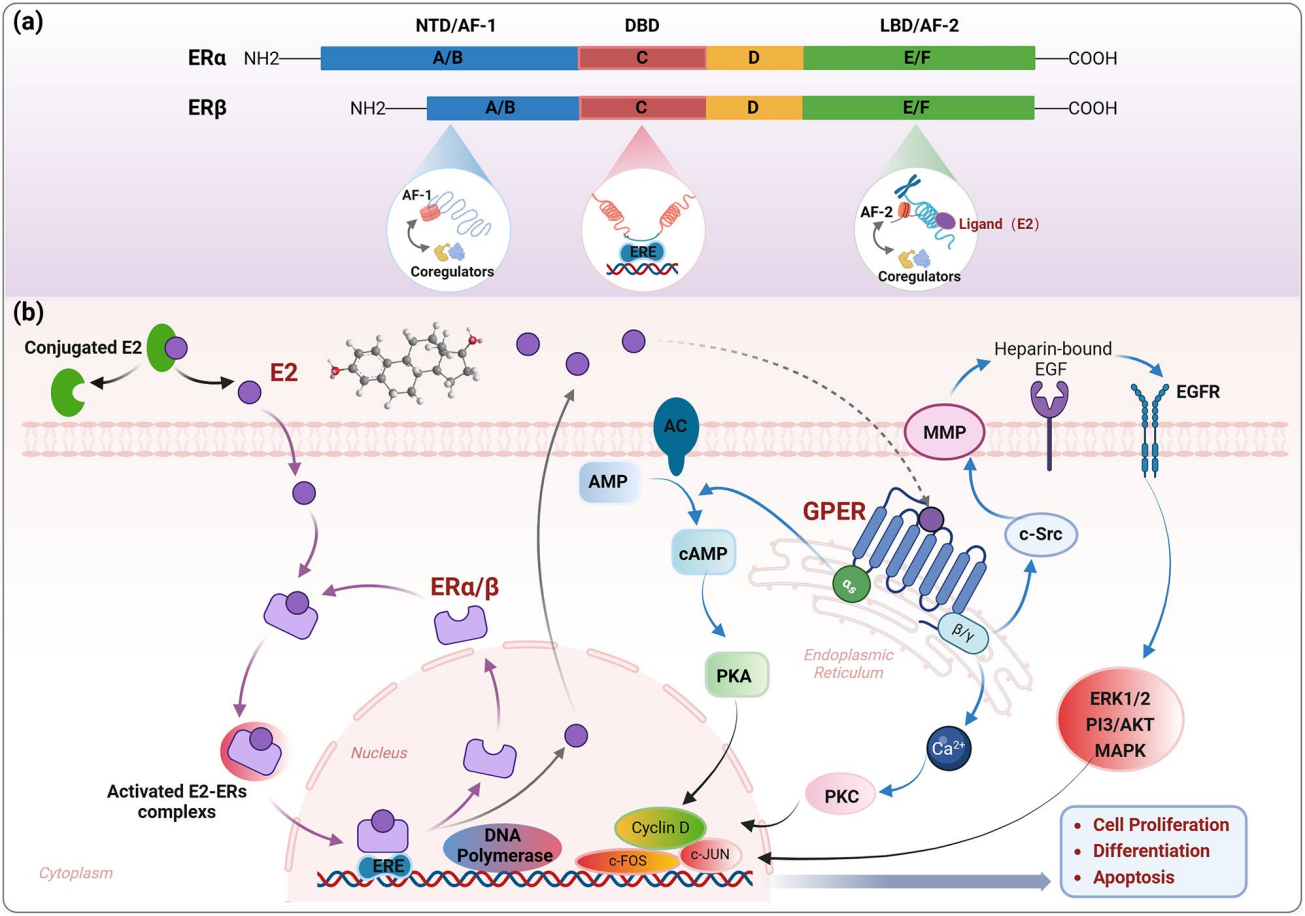
Structure and function of the estrogen receptors. (**a**) Structures of the ERα and ERβ isoforms. **(b)** E2 binds to ERα/β and GPER to activate genomic (purple arrows) and non-genomic signaling (blue arrows), respectively. *DBD* DNA binding domain, *E2* 17β-estradiol, *ERE* estrogen-responsive element, *ERs* estrogen receptors, *GPER* G protein-coupled estrogen receptor, *LBD* ligand binding domain, *MMP* matrix metalloproteinases, *NTD* NH2-terminal domain

Classical estrogen receptors (ERs) are categorized into two types, ERα and ERβ, serving as nuclear transcription factors that play a role in regulating various intricate physiological processes in humans. The ERα gene is situated on chromosome 6, while the ERβ gene is located on chromosome 14. Upon E2 stimulation, these receptors can activate distinct gene expressions [16]. Both ERα and ERβ comprise three functional domains: the NH2-terminal domain (NTD), the DNA-binding domain (DBD), and the ligand-binding domain (LBD), encompassing six regions (A-F) from the N- to the C-terminus [17] (Fig. 1a). The NTD, situated in the N-terminal A/B region, contains a ligand-independent activation function domain (AF-1) that plays a role in regulating the transcriptional activity of target genes. ERα and ERβ exhibit a 30% similarity in this region [18]. The DBD (C region) facilitates the specific binding of ERs to estrogen-responsive element (ERE) in the DNA helix, thereby regulating the expression of target genes [17, 18]. ERα and ERβ are highly conserved in this region, displaying a 97% homology [17]. The D structural domain comprises a series of amino acids that separate the DBD from the LBD, promoting post-translational modifications in ERs. The LBD, located in the C-terminal E/F region, includes a hormone-dependent AF-2, the hormone-binding cavity, and the dimerization interface. The LBDs of ERα and ERβ share a 59% similarity [19, 21]. Despite this, there are still minor structural discrepancies between the ligand-binding pockets of these two isoforms, primarily due to distinct amino acids in the ERα/β binding cavities [18].

The ligand-dependent pathway is one of the major mechanisms that regulates ERs through two distinct activation domains, AF-1 and AF-2 [19]. Typically, AF-1 and AF-2 work synergistically to control the transcriptional activity of ERs. AF-1 is notably highly expressed in ERα [18]. ERs modulate transcription by recruiting various transcriptional co-regulators (co-activators or co-repressors) that play crucial roles in activating or repressing target genes [19]. More than half of the transcriptional co-regulators differ between ERα and ERβ, potentially explaining the distinct roles of the two receptor subtypes [20]. Upon binding to E2 (ligand), ERs undergo conformational changes, detach from chaperone proteins, homodimerize, and bind to specific DNA response elements (ERE) near target genes [21, 22] (Fig. 1a, b). DNA-binding receptors interact with the transcriptional apparatus through cofactor proteins, leading to the expression of downstream target genes. However, the precise mechanism by which the activating functional domains of ERs regulate their transcriptional activity remains unclear [18]. Genetic studies on the DNA-binding domain have revealed significant overlap in the binding sites of ERα and ERβ, suggesting high similarity in this domain [17, 23]. Nevertheless, differences also exist in the DNA-binding domain of the two ERs. Gene expression analyses indicate that ERα upregulates genes associated with cell proliferation, while ERβ enriches genes controlling cell cycle progression and apoptosis [24, 25]. In breast cancer cells, ERβ suppresses around 70% of ERα-regulated genes linked to proliferation and metabolism [17]. These findings imply that structural and functional variances between ERα and ERβ underlie their activation of distinct genes and signaling pathways.

The expression levels of ERα and ERβ exhibit significant variation in normal intestinal epithelial cells, intestinal tumor cells, and across different tumor stages. ERβ is the predominant ER in colonic epithelium, with lower mRNA levels in CRC compared to normal tissues [16, 26]. Conversely, ERα is expressed at much lower levels than ERβ in normal colonic mucosal cells [27, 28]. Research indicates that ERα expression may act as a tumor promoter in the early stages of CRC, while ERβ tends to decrease during CRC progression [16, 29]. Clinical evidence shows that CRC tissues with high ERβ expression exhibit elevated levels of anti-tumor proteins like CysLT2R and 15-PGDH, alongside lower levels of pro-tumor proteins such as nuclear β-catenin, COX-2, and CysLT1R [26]. Patients with high ERβ expression and ERα negativity generally have improved overall survival, disease-free survival, and prognosis compared to those with low ERβ expression and ERα positivity in CRC [27]. Low ERβ levels are associated with local CRC recurrence, while high ERα levels may promote distant metastasis, particularly liver metastasis [26, 28]. Studies in *Apc*^Min/+^ mice and CRC cell lines have demonstrated the protective role of ERβ in CRC development, where ERβ deletion leads to increased colonic adenomas, while ERβ-specific agonists exhibit tumor-suppressive effects [30]. In contrast, ERα expression was elevated in mice with wild-type CysLT1R, and ERα-selective agonists promoted HT-29 and Caco-2 cell survival by activating the Wnt/β-catenin pathway. Inhibition or knockdown of ERα reduces its ability to promote cell survival and metastasis [28]. These findings provide evidence for the potential antitumor effects of ERβ in colorectal cancer (CRC), suggesting that the expression levels of ERα and ERβ could serve as biomarkers for assessing the risk and prognosis of CRC. While ERβ shows promise as a therapeutic target, conflicting reports on its expression and function in CRC exist. These discrepancies may stem from individual variations or a weak correlation between ERβ protein and mRNA levels. Thus, further comprehensive investigations are required to solidify the understanding of ERβ’s role in CRC.

### GPER

In addition to ERα and ERβ, GPER, also known as GPER1 and GPR30, is a membrane estrogen receptor that has attracted much attention in recent years [25].The human GPER gene is situated on chromosome 7, and GPER plays a role in the rapid signaling of estrogen and can indirectly enhance its transcriptional effects through multiple pathways [31]. GPER is localized on the endoplasmic reticulum and Golgi membranes, featuring seven transmembrane domains [31, 32]. It consists of 375 amino acids, with ligand binding and receptor activation primarily occurring in the N-terminal region; the C-terminal PDZ domain interacts with other plasma membrane proteins and determines the receptors’ placement on the cell membrane [18]. GPER transmits signals by interacting with various G proteins, including Gα_s_, Gα_i_, G_βγ_, and Gα_q/11_ proteins, forming heterotrimeric G proteins [33]. When GPER binds to Gα_s_, it directly activates adenylate cyclase, leading to the conversion of ATP to cAMP [34]. Binding of GPER to G_βγ_ prompts the release of intracellular calcium ions and activates matrix metalloproteinase (MMP), subsequently triggering the release of heparin-bound EGF from the outer plasma membrane. EGF then binds and activates the EGFR family, initiating downstream signaling through ERK1/2 [34]. Moreover, GPER’s non-genomic signaling in response to E2 can activate various signaling pathways such as PI3K/Akt, MAPK, and others, indirectly influencing transcriptional activity [31, 32] (Fig. 1b).

Clinical and experimental evidence regarding the pro- and anticancer activities of GPER is conflicting, making its role in CRC development controversial [35]. For instance, significant downregulation of GPER expression was observed in human CRC tissues, and Kaplan-Meier analysis indicated that high expression of GPER was associated with a favorable prognosis for CRC [36]. In contrast, analysis of The Cancer Genome Atlas dataset revealed that high expression of GPER was significantly associated with survival in female CRC patients at stage III-IV but not in female CRC patients at stage I-II or male CRC patients at any stage [35, 37–39]. Experimental data indicated that GPER expression was downregulated in HCT-8 and SW480 cells [36], while strong expression of both GPER mRNA and protein was detected in HT29, DLD1, SW620, and T84 cell lines [37]. Specific activation of GPER inhibits progression in the xenograft tumor model in vivo and suppresses CRC cell proliferation, initiates cell cycle arrest, induces endoplasmic reticulum stress, and mitochondria-associated apoptosis in vitro [36]. Additionally, GPER can inhibit the upregulation of the oncogene *JUN* and exert oncostatic effects by reducing the over-activated Wnt/β-catenin pathway in CRC [37]. At first glance, these results may seem contradictory. However, it is essential to note that Kaplan-Meier analyses performed on clinical tissues are observational, and a causal relationship cannot necessarily be inferred. Moreover, since these CRC cell lines originated from patients with differing genders, ages, tumor stages, or comorbidities, the GPER expression levels, GPER-mediated signaling pathways, and oxygen content in cells also varied [40]. Therefore, GPER expression in CRC cell lines is not entirely consistent with their tumorigenicity or metastatic potential.

## Precise molecular and cellular mechanisms by which E2 exerts its anti-CRC effects

### Enhancing DNA mismatch repair and regulating epigenetic events

The mismatch repair (MMR) system, encoded by MMR genes, maintains genomic stability by correcting base-pair mismatches that occur during DNA replication. Mutations in these genes often lead to microsatellite instability (MSI) [41], a hallmark of certain types of CRC characterized by an accumulation of mutations due to the failure of the MMR system, primarily involving the loss of MLH1, MSH2, MSH6 and PMS2 protein function [42]. In the functional state, MLH1 and MSH2 combine to form heterodimers known as MutLα and MutSβ, respectively, with PMS2 and MSH6. MLH1 and MSH2 act as chaperone proteins for their respective heterodimers. Mutations in the MLH1 or MSH2 genes can result in the degradation of these dimerized proteins, leading to the loss of essential proteins and chaperone proteins [41, 42]. Research indicates that E2 can induce the expression of MLH1 in both colon epithelial and breast cancer cells [43, 44]. In CRC cell lines, free E2 was found to increase MLH1 expression at both the mRNA and protein levels, while BSA-conjugated E2 had a lesser effect. This suggests that E2 regulates MLH1 expression through the typical ERs pathway [45]. Moreover, research has demonstrated that overexpression of ERβ can enhance MLH1 expression at both the mRNA and protein levels, whereas ERα does not impact MLH1 gene expression. Interestingly, treatment with E2 failed to induce MLH1 expression when ERα was overexpressed or ERβ was knocked down. Additionally, Western blotting analysis revealed that treating colorectal cancer cells with ERβ agonists significantly raised MLH1 protein levels [45]. Clinical data have also shown that the cytosine-adenine repeat genotype in the ERβ gene (ESR2) and resultant ERβ expression levels, along with estrogen activation, may influence MMR status in CRC [46]. These findings indicate that E2 specifically enhances MLH1 expression via ERβ rather than ERα. Upon E2 stimulation, the increased MLH1 activity leads to the formation of a functional complex with PMS2, known as MutLα, which plays a role in repairing mismatched bases and single nucleotide insertions [42]. In addition, E2 increases the sensitivity of CRC cells to the chemotherapeutic drug 5-FU and inhibits tumor proliferation in vivo and in vitro [45].

MicroRNAs (miRNAs) are small, non-coding RNAs that regulate various critical cellular processes, including cell differentiation, proliferation, apoptosis, cell cycle and inflammation [47]. Alterations in miRNA expression can lead to oncogenic or tumor-suppressive effects [48, 49]. E2 has been found to modulate the expression of specific miRNAs and MMR genes in CRC cells through ERβ [50]. In ERβ over-expression COLO205 cells, E2 reduced the expression of miR-135b, miR-31, and miR-155 while up-regulating hMLH1 expression in a time- and dose-dependent manner. Treatment of COLO205 cells with estrogen antagonists ICI182,780 reversed the E2-induced down-regulation of miRNAs and increase in hMLH1 expression. However, varying concentrations of E2 did not affect the expression of miRNAs or hMLH1 in SW480 cells with low ERβ expression [50]. Furthermore, E2 did not appear to influence other core MMR genes such as hMSH2. In human CRC tissues, miR-135b expression was notably higher in cancer tissues compared to normal tissues. A strong positive correlation between serum E2 levels above 45 pg/ml and hMLH1 expression was observed; whereas, no significant correlation was found when E2 levels were lower. Additionally, serum E2 levels showed a significant negative correlation with miR-31 and miR-135b expression in CRC tissues, and a positive correlation with ERβ, but not with miR-155 expression. Conversely, hMLH1 levels were negatively correlated with miR-155 and miR-135b expression in cancer tissues [50]. Studies have suggested that changes in the target binding sites of certain miRNAs in the MMR gene could potentially predict CRC risk and prognosis, and specific miRNAs may play a role in regulating the core heterologous proteins of the MMR [51–54]. These findings indicate that E2 down-regulates the expression of oncogenic miRNAs (especially miR-135b) through ERβ, potentially leading to the up-regulation of MMR activity. The differential effects of E2 on miRNA expression in CRC tissues or different cell lines appear to be associated with the level of ERβ.

Emerging evidence shows that the circadian system appears to influence cancer progression in a gender-dependent manner [55–58]. ERβ was found to be a regulator of the clock genes *bmal1* and *Npas2*, and a high-fat diet (HFD) altered the expression of key clock genes *bmal1* and *Npas2* in colon epithelial cells, with sex differences [58]. E2 can regulate HFD-induced alterations in core clock genes *via* ERβ, thereby inhibiting colon cell proliferation [58]. The clock gene *per2* is one of the target genes of the tumor suppressor miR-34a [59]. Recent reports have shown that miR-34a significantly inhibited the expression of clock genes (*per2* and *bmal1*) and clock control genes (*rev-ERβa*, *cyclin D1*, and *sirt1*) in DLD1 and LoVo cell lines, but did not affect the expression of ERβ [49]. While E2 reduced the proliferative and migratory activity of DLD1 cells, it did not significantly impact the expression of mature miR-34a. Interestingly, the combined administration of E2 and miR-34a did not synergistically enhance oncogenic effects, potentially due to miR-34a not influencing ERβ expression [49]. These findings indicate that E2’s inhibitory effect on CRC cells seems to be unrelated to the miR-34a-mediated pathway, despite its potential regulation of certain clock genes. In addition to regulating core clock genes, previous studies have identified cannabinoid receptor 1 (*CB1*) as an estrogen-responsive gene in CRC cell lines [60, 61]. E2 significantly inhibits the proliferation of CRC cell lines by activating the *CB1*-promoting region in exon 1 of the *CNR1* gene, which induces ERβ to bind to the *CNR1*-promoting region and upregulates *CB1* expression [60, 61].

**Fig. 2:**
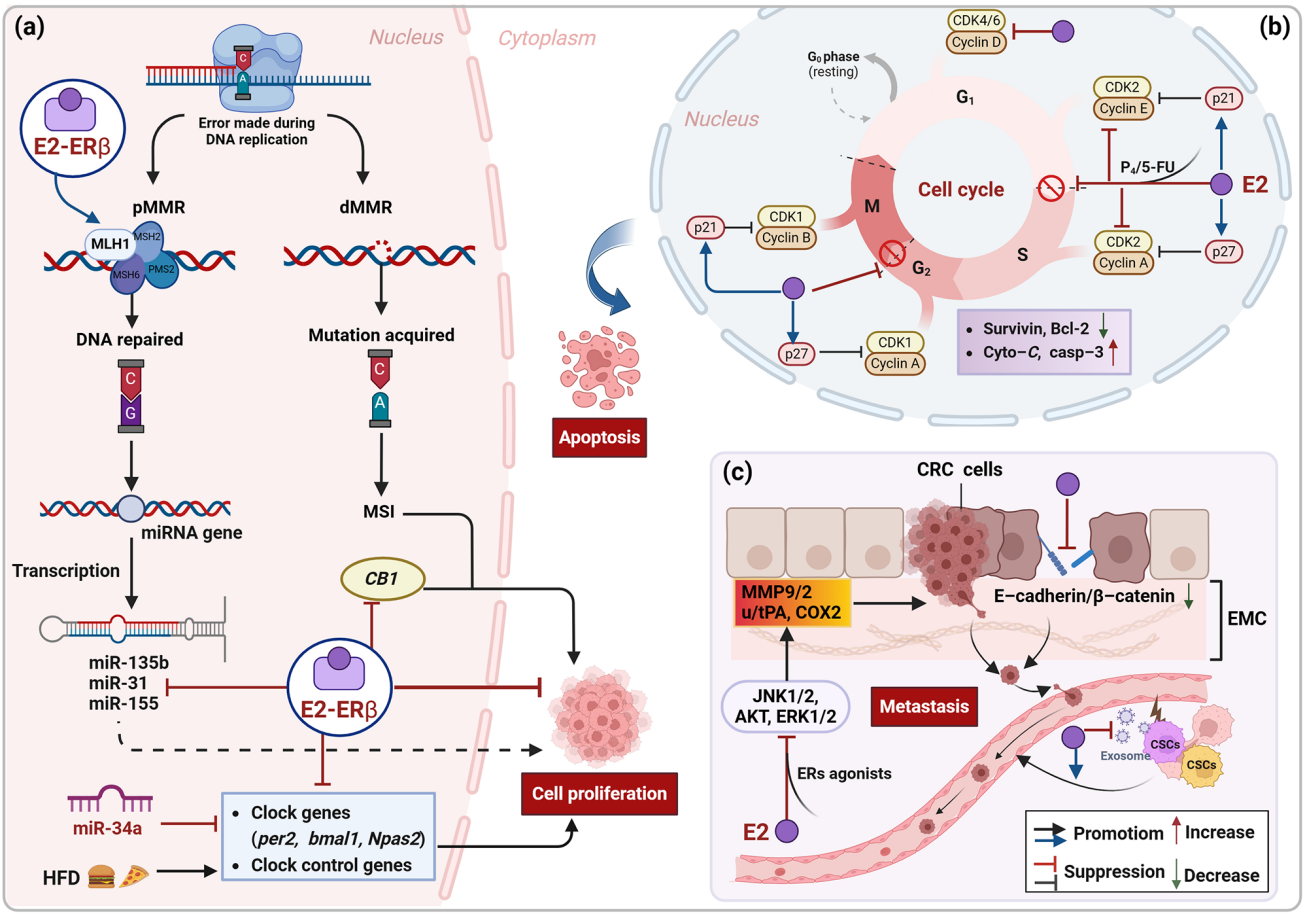
Precise molecular mechanisms by Which E2 inhibits CRC cells proliferation, migration and induces apoptosis. **(a)** E2 enhances DNA mismatch repair, regulates miRNA and core clock gene expression. **(b)** E2 initiates cell cycle arrest in sub-G1 phase and induces apoptosis. **(c)** E2 inhibits cell migration by downregulating the activity of MMPs and PAs and restoring the integrity of intercellular adhesions. However, E2 promotes CSCs migration by decreasing their affinity for endothelial cells on the surface of vascular lumen. *CSCs* cancer stem cells, *CRC* colorectal cancer, *E2* 17β-estradiol, *ECM* extracellular matrix, *ERs* estrogen receptors, *HFD* high-fat diet, *MMPs* matrix metalloproteinases, *MMR* mismatch repair, *dMMR* deficient MMR, *pMMR* proficient MMR, *MSI* microsatellite instability, *PAs* plasminogen activators

Taken together, at the genetic level, the protective effects of E2 on CRC are usually accomplished by increasing DNA mismatch repair or through epigenetic regulation mediated by miRNAs, core clock genes, and certain estrogen-responsive genes (Fig. 2a; Table 1).

### Triggering cell cycle arrest and inducing apoptosis

Studies have shown that in both male and female CRC cell lines, treatment with E2 alone or in combination with progesterone or 5-FU inhibits ERα expression while upregulating ERβ expression, promoting cell cycle arrest and apoptosis [62–64]. Genome-wide analysis showed that pathological processes like apoptosis, cell differentiation, and cell cycle regulation were significantly impacted in CRC cells re-expressing ERβ [54]. This aligns with previous research indicating that gene expression profiling of ERβ displayed an enrichment of genes that regulate cell cycle progression and apoptosis [24, 25]. E2 treatment alone or in combination with progesterone downregulated the expression of cell cycle sub-G1 phase markers CCND1/3 and cell survival marker survivin proteins by promoting the ERβ pathway. It also significantly increased apoptotic indices and p21, p27, cyto-C and casp-3 proteins expression in male-AOM model mice and human male CRC cell lines. Flow cytometry assays indicated that treatment with E2, either alone or in combination, led to a notable increase in the number of cells in the G0/G1 phase [62]. Studies by Mahbub et al. [63]. and Refaat et al. [64]. also demonstrated that E2 monotherapy significantly increased the number of male-SW480, female-HT29, and SW620 metastatic cells in the sub-G1 phase. These findings suggest that E2 prompts cell cycle arrest in the sub-G1 phase and initiates apoptosis by upregulating ERβ expression. Moreover, E2 alone or in combination with ERα-blockers also induced cell cycle arrest in the S- and G2/M-phases in male-SW480 cells, whereas it promoted only G2/M-phase arrest in female-HT29 cells. When combined with ERβ-blockers, E2 seemed to induce SW480 cells to arrest in the S-phase, with no effect observed on HT29 cells [64]. Furthermore, supplementation with ERβ-blockers inhibited, while ERα-blockers significantly enhanced, the pro-apoptotic effects of E2 [64]. These results suggest that ERα-blockers enhance the anticancer properties of E2, while ERβ-blockers diminish them. However, the impact of E2 on different cell cycle phases appears to be influenced by the cell source, particularly gender, and the expression status of ERs.

Dual treatment using E2 combined with 5-FU significantly induced apoptosis in male-SW480 and female-HT29 cells but had little effect in SW620 metastatic cells [63]. Furthermore, in terms of the period of intervention, E2 monotherapy could be more effective in treating advanced CRC, whereas the E2 plus 5-FU combination regimen represents a promising therapeutic strategy for early CRC [63]. Earlier studies also found that E2 reduced the expression of cell cycle regulatory proteins cyclin A, cyclin D1, cyclin E, PCNA, c-myb and Bcl-2 in a time-dependent manner, thereby inhibiting LoVo cell proliferation and promoting COLO-205 cell apoptosis [65, 66]. These findings suggest that E2 can induce cell cycle arrest and apoptosis in CRC cell lines (Fig. 2b; Table 1). However, the type of CRC cells, the concentration and duration of E2 treatment, and the drugs used in combination with E2 varied among the different literature. Therefore, these results do not fully account for the mechanism by which E2 exerts its protective effects.

### Inhibiting cell migration

In addition to its role in regulating the cell cycle and promoting apoptosis, Transwell migration assays demonstrated that E2, both alone and in combination with tamoxifen, reduced the viability and migratory capacity of DLD-1 cells, enhanced apoptosis, and diminished survivin protein expression in a dose- and time-dependent manner [67]. The degradation of the extracellular matrix (ECM) by extracellular proteases, leading to the release of growth factors and pro-angiogenic molecules, accelerates cancer cell invasion and migration [68]. MMPs and plasminogen activators (PAs) are the primary proteolytic systems responsible for ECM degradation [69, 70]. E2 significantly reduced LoVo cell migration by downregulating uPA, tPA, MMP-2 and MMP-9 expression through the activation of the E2/ERs-p38a MAPK and *p53* signaling pathways [65, 71]. Furthermore, ER antagonists completely negated E2’s inhibitory effects, while ER agonists, in conjunction with E2, further reduced the activity of cell migration factors [65, 71]. Notably, ERβ isoforms play a pivotal role in the suppression of migration-associated proteins mediated by E2/ERs or ER complexes [65]. E2 also markedly inhibited prostaglandin E2-induced LoVo cell migration. This effect was achieved by suppressing the activities of uPA, MMP-9, and COX-2 through the inhibition of the JNK1/2, Akt, and ERK1/2 pathways [72, 73] (Fig. 2c). However, these studies have limitations, such as their exclusive focus on the impact of E2 on the migratory activities of LoVo and DLD-1 colon cancer cells without observing other CRC cell lines. Therefore, these findings may not be applicable to all CRC cell lines.

Normal enterocyte migration is usually accompanied by a switch to a functional cell state dependent on the formation of cell-cell contacts mediated by cadherin [74]. A previous study concluded that E2 improves crypt-villus migration of enterocytes in *Apc*^*Min/+*^ CRC model mice, which plays an important preventive role in the early stages of intestinal carcinogenesis [75]. Mechanistically, E2 enhanced the association between E-cadherin and β-catenin in the intestinal mucosa and induced E-cadherin expression on the lateral cell membranes of enterocytes. This contributed to restoring the integrity of the lateral membrane adherens junctions and normalizing defects in intercellular adhesion and cell migration [75]. While E2 appears to play a protective role in early intestinal tumor formation by promoting enterocyte migration, it produces the opposite effect in colon cancer stem cells (CCSCs). A recent study showed that E2 had no effect on the viability and proliferation of CCSCs but inhibited exocytosis, exosome biogenesis, and enhanced cell migration [76]. Mechanistically, E2 upregulated both pro- and anti-apoptotic factors in CCSCs, stimulated the expression of homologous ERs and SQSTM1 protein, and inhibited the expression of SIRT1 and exosome biogenesis-related genes. Additionally, E2 reduced the affinity of CCSCs for endothelial cells on the surface of the vascular lumen, leading to CCSC migration [76] **(**Fig. 2c).

In summary, E2 can resist the proliferation, invasion and migration of CRC cells mainly by enhancing DNA mismatch repair, regulating miRNA and core clock gene expression, initiating cell cycle arrest in the sub-G1 phase, inducing apoptosis, and inhibiting the activity of cell migration factors (Fig. 2; Table 1). However, these observations require further validation in future studies due to variability in study methods, the CRC cell types and different E2 treatment concentrations and duration used.

## Effects of E2 signaling on colorectal tumor microenvironment

**Fig. 3:**
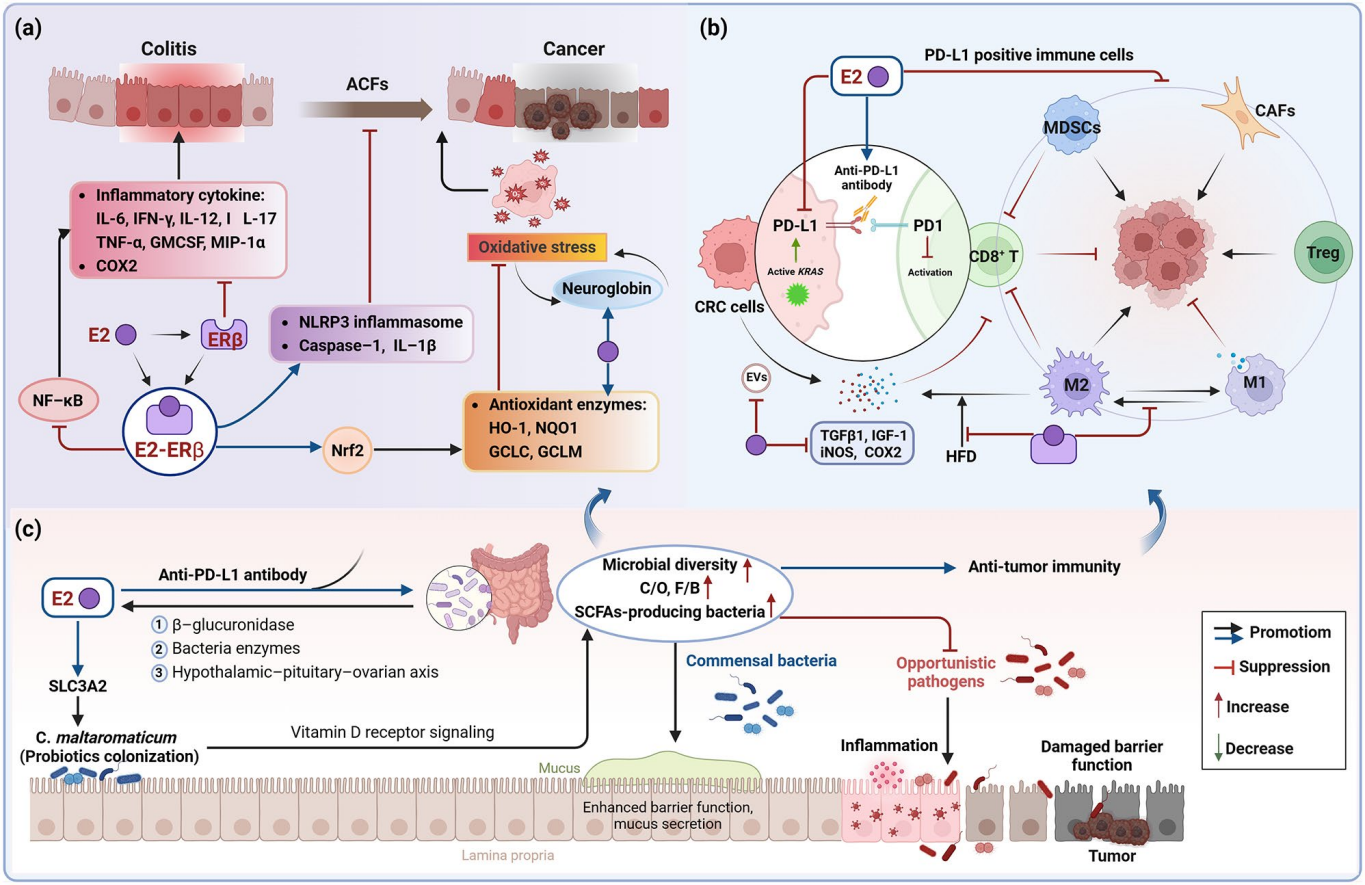
Effects of E2 signaling on the colorectal TME, i.e., inflammation, immunity, and the gut microbiome. **(a)** E2 inhibits early CRC progression by attenuating the inflammatory response, reducing ACFs formation and oxidative stress injury *via* ERβ. **(b)** E2 reverses immunosuppressive microenvironment chiefly through downregulating PD-L1 expression and regulating the ratios of infiltrating immune cells and tumor-associated cell populations. **(c)** E2 may alter the gut microbiome composition and diversity by modulating C/O ratio, F/B ratio, enhancing probiotics intestinal colonization, and increasing the abundance of SCFAs-producing bacteria, thereby maintaining the integrity of the intestinal barrier and reducing the risk of CRC. The gut microbiota, in turn, can influence E2 levels through multiple pathways. *CAFs* cancer-associated fibroblasts, *CRC* colorectal cancer, *C/O* commensal bacteria/opportunistic pathogens, *E2* 17β-estradiol, *ERβ* estrogen receptor beta, *EVs* extracellular vesicles, *F/B Firmicutes*/*Bacteroidetes*,* HFD* high-fat diet, *IGF-1* insulin-like growth factor 1, *MDSCs* myeloid-derived suppressor cells, *PD-L1* programmed death-ligand 1, *SCFAs* short chain fatty acids

Above, we discussed the molecular mechanisms at the cellular level, and now, we summarize the impacts of E2 treatment on the colorectal TME in the following sections, specifically focusing on early lesions, inflammation, immunity, and the gut microbiome (Fig. 3; Table 1).

### Stimulating ERβ expression and reducing inflammatory response

Previous studies have demonstrated that E2 inhibits growth and promotes apoptosis in non-cancerous young adult mouse colonocytes (YAMC), yet exhibits no effect on the isogenic YAMC-Ras cell line, which undergoes malignant transformation [77]. At the level of ovariectomized (OVX) animals, E2 treatment in both wild-type (WT) and ERβ knockout (ERβ_KO) mice resulted in fewer aberrant crypt foci (ACFs) and increased apoptotic activity in the colonic epithelium of WT mice [77]. In the OVX_AOM/DSS model, initiating E2 treatment after DNA damage onset was found to reduce colon tumor formation [78, 79]. However, E2 treatment did not influence the proliferation and apoptosis of colon tumors or undamaged colon crypt cells in ERβ_KO mice. Additionally, while ERβ is highly expressed in normal colon cells and promotes apoptosis, its expression decreases as colon epithelial cells transition from a non-malignant to a cancerous state, accompanied by an increase in ERα expression [78]. The presence of E2 did not alter the reduction in ERβ expression. Conversely, an earlier study indicated that E2 could induce short-term translation and late transcriptional enhancement of ERβ mRNA in DLD-1 cells *via* activation of p38/MAPK signaling [80]. These observations suggest that E2 exerts a protective effect in the early stages of CRC development but depends on the presence of ERβ.

E2 was reported to exhibit varied protective impacts against chemical carcinogen-induced acute colitis and colon tumor formation, influenced by the presence of ERβ [81]. These protective effects were different between ERβ_KO and WT mice concerning the inflammatory factor expression profile and the injury protection site. After E2 treatment, reductions in IL-6 and IFN-γ levels were observed in both groups, but in WT mice, the levels of IL-12/17, TNF-α, GMCSF, and MIP-1α also decreased. The site of inflammatory injury alleviation varied, with ERβ_KO/E2 mice showing improvements primarily in the distal colon and WT/E2 mice in the mid-colon. Additionally, ERβ_KO/E2 mice exhibited increased crypt proliferative activity in the distal colon’s one-third and decreased apoptosis in the proximal colon. Following E2 administration, no proliferative alterations were noted in the proximal colon crypts of either group [81]. Similarly, E2’s protective role against colitis and proximal colon carcinogenesis was validated in the OVX_AOM/DSS model. E2 supplementation markedly lowered the incidence of proximal colon tumors prompted by OVX [79]. Mechanistically, E2 mitigated colitis-associated cancer (CAC) by diminishing ERα and NF-κB signaling pro-inflammatory markers (such as COX-2, TNF-α, and IL-6) and by increasing the expression of ERβ, Nrf2 and related antioxidant enzyme genes (like HO-1, NQO1, GCLC, and GCLM) [79, 82] (Fig. 3a). E2’s anti-inflammatory action in early colitis stages is facilitated through Nrf2; nonetheless, this effect alone may not prevent distal colon tumor development. Conversely, in the absence of Nrf2, E2 significantly obstructed distal colon tumor development *via* ERβ-dependent pathways [83]. Additionally, E2 notably elevated the expression of NLRP3 inflammasome, along with its associated phosphorylated caspase-1 and IL-1β in AOM/DSS mice [82]. At the cellular level, E2 suppressed the inflammatory response in female human colonic epithelial CCD841CoN cells by reducing NF-κB and COX-2 expression and by upregulating the antioxidant enzymes HO-1 and NQO-1. Conversely, E2 treatment increased ERβ expression but did not affect ERα in CCD841CoN cells [84]. These findings indicate that mitigating chronic intestinal inflammation by inhibiting inflammation-associated signaling and enhancing antioxidant enzyme expression could be among the mechanisms through which E2 lowers CAC risk (Fig. 3a).

Pro-inflammatory substances produced by oxidative stress can trigger inflammatory response. Along with upregulating the expression of antioxidant enzymes to reduce oxidative stress damage, ERβ-mediated E2 activation can enhance neuroglobin expression in DLD-1 cells. Neuroglobin serves as an oxidative stress sensor and facilitates E2-induced apoptosis in the absence of oxidative stress. Conversely, under conditions of high oxidative stress (100 µM H_2_O_2_), neuroglobin interacts with cytochrome *c*, inhibiting its release into the cytoplasm and preventing the ensuing apoptotic process, thus obstructing E2’s pro-apoptotic effects [85]. This finding suggests that the upregulation of neuroglobulin in CRC cells may serve as a defense mechanism against oxidative stress damage. Furthermore, this defense mechanism appears to counteract the antioxidant effects of E2. While many studies have verified E2’s protective role against colitis and CRC, contrasting evidence exists. A specific study found that E2 facilitated intestinal tumor growth in the context of inflammatory injury [86]. E2 administration notably exacerbated colitis-related symptoms and increased the number of polyps in OVX_AOM/DSS mice, significantly driving the development and progression of invasive adenocarcinoma. At the molecular level, E2 substantially boosted Il-6 production and the count of Ki67-positive cells while reducing the number of active caspase-3-positive cells (indicative of apoptosis). Additionally, the pro-tumorigenic effects of E2 were found to be contingent upon both ERα and ERβ, with ERα exacerbating inflammation and ERβ promoting tumorigenesis in a manner not directly linked to inflammation [86]. However, the study did not extensively investigate the specific pathways through which ERβ facilitates tumorigenesis, and the available evidence is insufficient to conclusively support its contentious findings.

Collectively, maintenance of ERβ expression is one of the most important mechanisms by which E2 plays a protective role in CRC. E2 typically hinders early CRC progression by decreasing the inflammatory response, lowering the formation of ACFs, and reducing oxidative stress through ERβ activation (Fig. 3a). However, in cases where there is pre-existing inflammatory damage, the expression levels of ERs may change, leading E2 to potentially have pro-inflammatory effects. Therefore, administering E2 before the onset of inflammation is more beneficial for it to exert its anti-inflammatory properties.

### Modulating immunosuppressive microenvironment

Previous studies have indicated that E2 may protect normal intestinal epithelial cells from inflammation-induced carcinogenesis by enhancing the expression of Nrf2 and associated antioxidant enzymes, thereby maintaining redox homeostasis [79, 82]. In contrast, other research has shown that knocking down Nrf2 enhances the anti-tumor immune effects of E2 [83, 87]. AOM/DSS treatment led to significant upregulation of programmed death-ligand 1 (PD-L1) in colonic tissues of both WT and Nrf2_KO male mice [87]. Expression of PD-L1 in tumor cells or tumor-infiltrating immune cells reduces T cell activation, inhibiting T cell cytotoxicity and the resulting anti-tumor immune responses [88]. Additionally, E2 supplementation significantly decreased PD-L1 expression as well as iNOS and COX-2 levels in Nrf2_KO AOM/DSS male mice [87]. These pro-inflammatory factors are known to induce PD-L1 expression in tumor cells and attract PD-L1-positive immune cells to the tumor site, contributing to an immunosuppressive TME [87]. In the OVX_MC38 colon tumor model, the population of cytotoxic CD8^+^ T cells was found to be lower, while the population of PD-L1-positive M2-like macrophages, myeloid-derived suppressor cells (MDSCs), and Treg cells was significantly higher [89]. This indicates that the tumor immune microenvironment (TIME) is suppressed following estrogen deprivation, and this suppression can be reversed with E2 supplementation. However, in the CRC liver metastasis model, E2 was found to promote the accumulation of TNFR2, F4/80^+^ tumor-associated macrophages (TAMs), and MDSCs in the hepatic immune microenvironment. It also decreased the proportion of CD8^+^ T cells, ultimately leading to the promotion of liver metastasis [90]. Notably, varying concentrations of E2 did not influence cell proliferation or apoptosis in vitro, nor did it alter PD-L1 expression in MC38 cells [89]. Further investigations revealed that most extracellular vesicles (EVs) in MC38 colon tumors originated from the tumor cells themselves rather than immune cells. These EVs contributed to establishing an immunosuppressive TME. E2 supplementation diminished the level of the immunosuppressive factor TGF-β1 in EVs and its capacity to induce Treg cells, suggesting E2 might indirectly hinder MC38 tumor growth by modulating the TIME *via* MC38-derived EVs [89]. In this model, male mice demonstrated accelerated tumor growth, elevated PD-L1 protein levels in MC38 tumor sections, and higher proportions of PD-L1-expressing tumor cells, M2-phenotyped TAMs, and cancer-associated fibroblasts (CAFs) compared to female mice [91]. Moreover, combining E2 with anti-PD-L1 treatment synergistically reduced PD-L1 expression in tumor tissues, diminished the population of PD-L1-expressing tumor cells and TAMs (CD11b^+^F4/80^+^), and significantly inhibited MC38 tumor growth in male mice. This study also observed that E2 treatment prior to MC38 cell injection significantly decreased tumor weight, whereas post-injection E2 treatment had no impact on tumor size [91], suggesting the timing of E2 intervention is crucial for its effectiveness in inhibiting tumor growth. These findings align with prior research indicating that E2’s effects on CRC, whether protective or pro-metastatic, are primarily mediated through modifications in the colonic immune microenvironment rather than direct actions on MC38 tumor cells [89–91]. Collectively, these findings suggest that E2 indirectly inhibits the progression of CRC by modulating the immune microenvironment. This is primarily achieved through reducing PD-L1 expression and adjusting the populations of infiltrating immune cells like TAMs, MDSCs, Treg, CD8^+^ T cells, and CAFs within the tumor (Fig. 3b). Notably, E2 appears to have contrasting effects on the immune microenvironments of the intestines and liver, possibly due to distinct compositions and functions within their microenvironments.

Obesity is recognized as a significant risk factor for CRC, particularly in men, and this association appears to be linked to estrogen. Recent studies have demonstrated that HFD-fed mice had dysregulated metabolism, increased macrophage infiltration, enhanced proliferation of colonic crypts, and accelerated tumor growth [58, 92]. In models of HFD-fed MC38 tumor-bearing mice, OVX_females had greater inflammation associated with macrophages in subcutaneous adipose tissue, elevated levels of insulin-like growth factor 1 (IGF-1), and a higher prevalence of M2-like TAMs compared to their female counterparts [92]. Further analysis of TAM gene expression in OVX mice revealed an upregulation of the M2-like macrophage marker (CD206) and a significant downregulation of the M1-like macrophage marker (CD11c) [92], indicating that the absence of estrogen promotes the development of tumor-favoring macrophages in OVX_MC38 mice. Moreover, E2 was found to counteract HFD-induced infiltration of F4/80^+^ macrophages and proliferation of colon epithelial cells in male mice through ERβ while concurrently decreasing body weight and enhancing the metabolic profile of the colon *via* ERα [58]. These findings suggest that estrogen signaling can mitigate some of the adverse effects induced by HFD on the colonic immune microenvironment. Therefore, obesity, macrophage-associated inflammation, and TAMs constitute potential pathways through which CRC develops in both men and women with estrogen deficiency (Fig. 3b). This knowledge offers valuable insights for the prevention and management of obesity-related CRC.

### Regulating the gut microbiome composition and diversity

In addition to attenuating intestinal inflammation and modulating the immunosuppressive microenvironment, E2 also regulates the composition and function of the gut microbiome in CRC model mice. This novel research area, termed the “microgenderome,” explores the interplay between sex hormones, primarily E2, and the gut microbiome [93, 94], highlighting its significance in CRC development. Prior investigations have demonstrated E2’s capacity to suppress PD-L1 expression in immune cells infiltrating tumors and its synergistic effect with anti-PD-L1 antibodies in curtailing the growth of MC38 colon tumors [89, 91]. Recent findings shed light on the gut microbiome’s response to treatment with E2 alone or in combination with anti-PD-L1 antibodies in MC38 mice [95]. The studies revealed that treatment with either E2 or anti-PD-L1 influenced the β-diversity of the microbiota in both male and female mice, although the alterations were not statistically significant. Treatment with anti-PD-L1 alone was observed to enhance the presence of the commensal bacterium *Parabacteroides goldsteinii* in both genders. Moreover, the combined therapy of E2 and anti-PD-L1 increased the abundance of *Parabacteroides goldsteinii* and the *Lactobacillus murinus* group in male mice while concurrently reducing the presence of opportunistic pathogens from the *Enterobacteriaceae* family. E2 treatment alone or in combination with anti-PD-L1 usually modulates four microorganisms, resulting in an increase in the family *Ruminococcaceae* (PAC001785 and PAC001716) and a decrease in the family *Muribaculaceae* (PAC001070 and PAC00106). No significant impact of E2 treatment or sex differences was observed on the abundance ratio of the two major phyla, *Firmicutes*/*Bacteroidetes* (F/B). In the context of the MC38 colon tumor model, E2, whether applied alone or combined with anti-PD-L1, led to an increased F/B ratio in males [95]. Conversely, in the AOM/DSS model, E2 supplementation markedly decreased the F/B ratio and modified microbial diversity (Chao1, Shannon, and Simpson indices) in male mice [96]. Furthermore, while the relative abundance of both commensal bacteria (PAC000664 and *Phocea*) and opportunistic pathogens (*Pseudoflavonifractor* and *Neglecta*) decreased in male mice treated with AOM/DSS and E2, the ratio of commensal bacteria to opportunistic pathogens increased [96]. These findings indicate that E2 may influence the composition of the gut microbiome, increasing the ratio of commensal bacteria to opportunistic pathogens and adjusting the F/B ratio, potentially reducing CRC risk and bolstering anti-tumor immunity (Fig. 3c). It is important to acknowledge that the literature predominantly examines the MC38 and AOM/DSS CRC models, and the relative abundance and ratio of gut flora are subject to dynamic changes over time and space. As such, the apparent discrepancies in the effects of E2 on F/B ratios may reflect differences between animal models.

*Carnobacterium maltaromaticum* has been reported to be specifically absent in the feces of female CRC patients [97]. In both *Apc*^Min/^+ and AOM/DSS CRC model mice, treatment with *C. maltaromaticum* significantly reduced the size and number of intestinal tumors in female mice but not in male mice [97]. To investigate the association between estrogen and the anticancer effects of *C. maltaromaticum*, researchers assessed the intestinal tumor formation in OVX_mice receiving *C. maltaromaticum* with or without E2 supplementation and found that E2 supplementation restored the anticancer effects of *C. maltaromaticum.* A similar phenomenon was also observed in ORX_male mice supplemented with E2 and *C. maltaromaticum.* At the cellular level, E2 significantly increased the attachment of *C. maltaromaticum* to colonocytes NCM460 but did not affect cell viability [97]. Mechanistically, E2 enhances *C. maltaromaticum* attachment and intestinal colonization by upregulating colonic SLC3A2 expression. *C. maltaromaticum* treatment alters the composition of the intestinal microbiota, i.e., significantly enriched butyrate-producing bacteria *Faecalibacterium prausnitzii* and *Lachnispiraceae bacterium*, whereas reduced the abundance of opportunistic pathogens *Bacteroides vulgatus* and *Muribaculum intestinale*. These beneficial flora directly convert *C. maltaromaticum*-derived 7-DHC into downstream vitamin D metabolites and activate vitamin D receptor signaling, thereby maintaining intestinal barrier function and reducing mucosal inflammation [97]. Overall, *C. maltaromaticum* colonizes the intestinal tract in an E2-dependent manner and, together with other microbiome to activate the vitamin D receptor signaling to inhibit CRC. As mentioned previously, E2 attenuates intestinal inflammation and inhibits CAC mainly through ERβ [79, 81, 82, 84]. It has been found that AOM/DSS treatment combined with ERβ knockdown facilitates the enrichment of microbiota affecting cell motility and carbohydrate metabolism, and reduces the gut microbiota diversity [98]. This suggests that intestinal ERβ contributes to a more favorable microbiome and assists E2 in delaying the progression of CAC. A recent study reported that zearalenone, an estrogenic mycotoxin, increased the abundance of short chain fatty acids (SCFAs)-producing bacteria (*unidentified Ruminococcaceae*, *Blautia*, and *Parabacteroidies*), which favorably inhibited the development of CRC [99]. These findings suggest that estrogens may exert anti-CRC effects by enhancing certain probiotics intestinal colonization, increasing the abundance of SCFAs-producing bacteria, or altering the composition of the intestinal flora through ERβ-mediated pathways (Fig. 3c).

E2 significantly influences the composition and diversity of the gut microbiota, which in turn plays a crucial role in the regulation of E2 metabolism and cycling, thereby affecting E2 levels. This bidirectional interaction is termed the “sex hormone-gut microbiome axis“ [100–102]. E2 is known to increase the abundance of bacteria that produce SCFAs, supporting intestinal barrier integrity and improving energy metabolism by dampening inflammatory signaling pathways [103, 104]. Moreover, E2 mitigates the risk of malignant transformation from chronic enteritis by reducing the abundance of *Proteobacteria* as well as lipopolysaccharide synthesis, partly through the upregulation of intestinal alkaline phosphatase activity, an antimicrobial peptide [105]. Additionally, E2 modulates the host immune response by influencing the metabolism of the gut microbiota *via* ERs present in immune cells [106]. Conversely, the gut microbiome regulates host E2 levels through various mechanisms. One pathway involves the deconjugation of conjugated E2 by β-glucuronidase, producing biologically active free E2 that is reabsorbed in the enterohepatic circulation [107]. Alterations in gut microbiota composition can modulate β-glucuronidase activity, impacting the availability of free E2 and its subsequent physiological effects [103, 108]. Furthermore, the gut microbiota may harbor specific bacteria endowed with enzymes such as steroid reductase, hydroxysteroid dehydrogenase, and 17, 20 lyase, which are conducive to E2 biosynthesis [109, 110]. Additionally, the gut microbiome is implicated in regulating the release of neuromodulators that act on the hypothalamic-pituitary-gonadal axis, directly influencing ovarian function and endogenous E2 production. This complex interplay underscores the integral role of the sex hormone-gut microbiome axis in maintaining homeostasis and its potential impact on health and disease [111] (Fig. 3c).

Taken together, E2 regulates the composition and diversity of the gut microbiota, which in turn can influence E2 levels through multiple pathways. The bidirectional interactions between the two are important in the pathogenesis and treatment of CRC, suggesting that combination therapies targeting E2 and the gut microbiome could have promising potential to improve the prognosis of CRC patients.

## Non-genomic signaling of GPER-mediated E2 in CRC

**Fig. 4:**
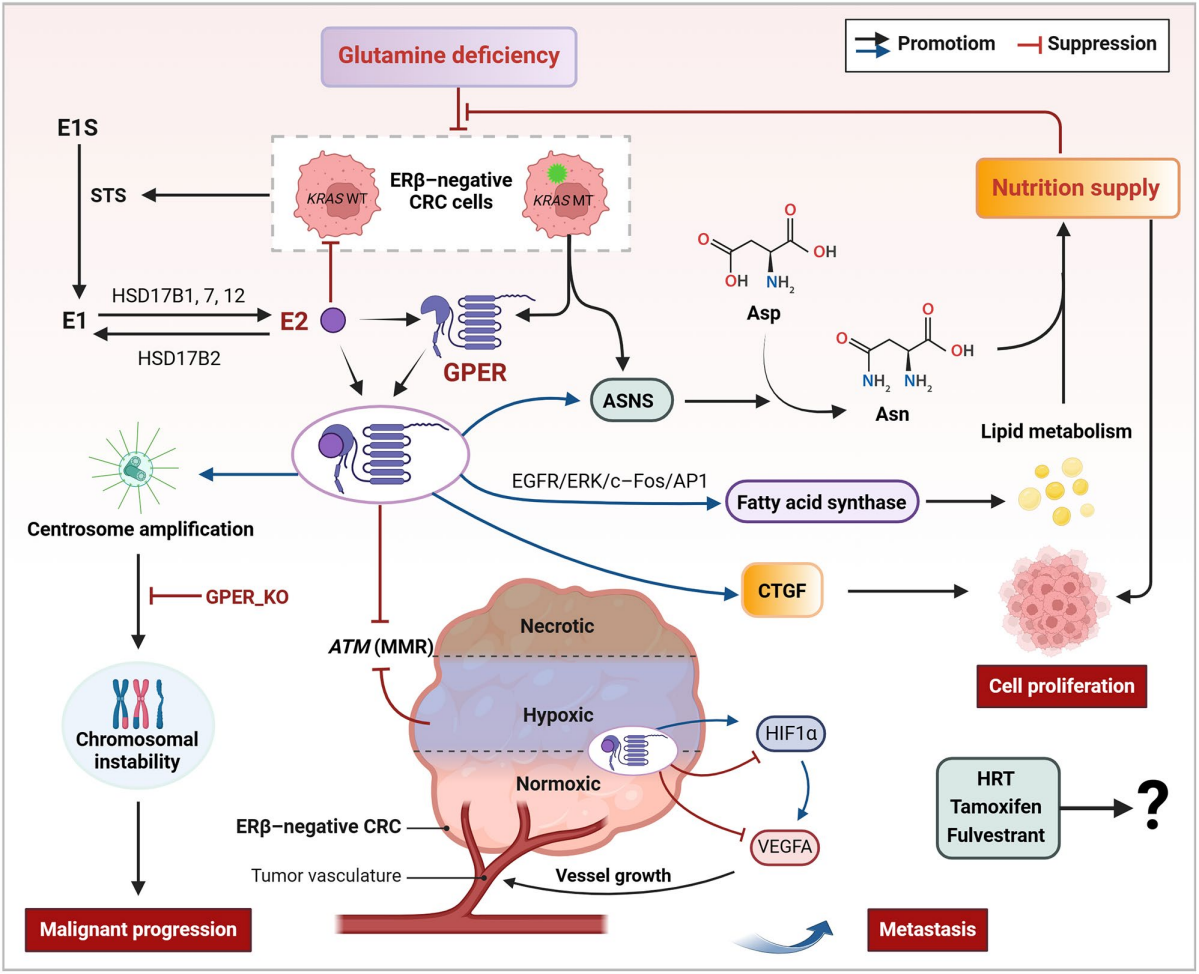
Non-genomic signaling of GPER-mediated E2 in CRC. In CRC cells that have lost ERβ expression or contain *KRAS* MT, E2 interacts with GPER to inhibit cancer progression under normoxic conditions. However, this interaction initiates metabolic reprogramming and shifts towards promoting tumor growth under conditions of hypoxia and nutrient deficiency. E2-stimulated GPER activation can lead to centrosome amplification, increased STS activity, upregulated oncogene expression, and the activation of lipid metabolism pathways, ultimately promoting cell proliferation and metastasis. *ASNS* asparagine synthase, *Asn* asparagine, *Asp* aspartate, *CRC* colorectal cancer, *CTGF* connective tissue growth factor, *E*_*1*_*S*, estrone sulfate, *E2* 17β-estradiol, *ERβ* estrogen receptor beta, *GPER* G protein-coupled estrogen receptor, *HRT* hormone replacement therapy, *MMR* mismatch repair gene, *MT* mutant, *STS* steroid sulfatase, *WT* wild-type

E2 primarily exerts its anti-CRC activity through ERβ; however, as CRC progresses, ERβ expression is gradually lost in the hypoxic microenvironment [38]. G protein-coupled ER (GPER, also known as GPER1) remains expressed after the loss of ERβ and thus becomes an important mediator of E2’s action in CRC [112]. In ERα/β-negative CRC cells, it has been observed that E2 can inhibit *ATM* (DNA repair gene) expression under both normoxic and hypoxic conditions *via* GPER [38]. Hypoxia triggers the activation of the hypoxia-inducible factor (HIF) pathway and subsequent downstream GPER signaling [113]. In normoxic conditions, E2 hinders the activation of HIF1α and vascular endothelial growth factor (VEGFA) in ERs-negative HT-29 cells, thereby suppressing angiogenesis. In contrast, under hypoxic conditions, E2 treatment enhances the hypoxia-induced activation of HIF and GPER signaling, resulting in the upregulation of VEGFA and HIF-1α expression, as well as promoting cell proliferation and migration. Knocking down GPER weakens E2’s inhibitory effect on VEGFA and HIF-1α, indicating its dependency on GPER [38]. These findings suggest that GPER mediates the anticancer or pro-oncogenic effects of E2 in ERs-negative CRC, with the former occurring under normoxic conditions and the latter under hypoxic conditions (Fig. 4). The oxygen tension within the TME might play a regulatory role in the interaction between E2 and GPER, with oxygen levels varying according to the CRC stage and tumor biopsy location. This variability could help clarify the complex dual nature of estrogen’s impact on CRC, swinging between tumor-suppressive and tumor-promoting actions. Beyond oxygen levels, the nutritional status within the TME is crucial for tumor cell proliferation. Using a 3D organoid model with *KRAS* mutant (MT) and *KRAS* wild type (WT) CRC cells, it was observed that glutamine deficiency curtailed the growth of both *KRAS* MT and *KRAS* WT cells and triggered an increase in GPER1 and asparagine synthase (ASNS) expression in *KRAS* MT cells. While E2 supplementation further inhibited the growth of *KRAS* WT cells, it did not affect *KRAS* MT cells. Additionally, E2 did not influence the upregulation of GPER1 and ASNS expression induced by glutamine deficiency [35]. Mechanistically, *KRAS* MT cells adapt to a reduced nutrient supply by elevating GPER1 and ASNS expression, which in turn supports tumor proliferation. Given that advanced tumors often experience nutrient scarcity, high levels of GPER1 and ASNS are linked with worse outcomes in advanced female CRC patients [35]. These findings imply that *KRAS* MT CRC cells may evade the protective effects of E2 through GPER1 under nutrient-poor conditions, positioning GPER1 and ASNS as potential therapeutic targets for *KRAS* MT CRC (Fig. 4). Notably, E2 did not alter GPER expression induced by nutritional deficiency, nor did it significantly affect GPER expression in CRC cells under normal nutritional conditions [114].

Excessive centrosome replication is closely linked with the onset, progression, and treatment resistance of CRC. A recent study found that GPER1 activated by high concentrations of E2 (10 nM) induces excessive centrosome replication and amplification in CRC cells. This results in the transient production of multipolar mitotic spindles, as well as alignment-defective and lagging chromosomes [115]. The knockdown or inhibition of GPER1 led to a reduction in the number of centrosomes and an increase in karyotype stability in CRC cells exposed to E2. Furthermore, the application of E2 or other GPER1 activators or inhibitors did not affect CRC cell proliferation, suggesting that GPER1’s role in regulating the number of centrosomes in CRC cells is distinct from its role in cell proliferation [115]. These findings underscore the essential role for E2-activated GPER1 in the malignant progression of intestinal susceptibility lesions. Increased expression of STS is also considered an indicator of poor prognosis in CRC. In CRC tissues and cells (HCT-116 and HT-29), the expression levels of STS, HSD17B7, and HSD17B12 were found to be elevated, while the expression of HSD17B2 was significantly reduced. This suggests that CRC upregulates the pathway that facilitates the desulfation of estrone sulfate (E_1_S) and the subsequent E2 synthesis [39, 116]. In HCT-116 and HT-29 cells, which lack ERβ and have low expression of HSD17B2, E2-stimulated activation of GPER promoted the expression of connective tissue growth factor (CTGF), thereby driving cell proliferation [39, 116]. In contrast, Caco2 cells, also ERβ-negative but with high HSD17B2 expression, did not respond to E2 stimulation, suggesting that HSD17B2 influences the local availability of E2, thus affecting the cancer cells’ ability to respond to E2-induced proliferation [39]. Interestingly, tamoxifen and fulvestrant, which are GPER agonists, also significantly increased STS activity in CRC cells [116]. These findings indicate that E2, GPER, STS, HSD17B2, HSD17B7, and HSD17B12 may be potential therapeutic targets for CRC. However, they also suggest that HRT, mainly consisting of estrone sulfate (E_1_S), tamoxifen and fulvestrant, could adversely affect CRC prognosis. Therefore, caution should be exercised when using these drugs in patients at high risk for CRC. Activation of lipid metabolism is a significant metabolic alteration in cancer cells. An early study discovered that E2 induces fatty acid synthase expression in ER-negative CRC cells and CAFs through GPER-mediated activation of the EGFR/ERK/c-Fos/AP1 signaling pathway, stimulating cancer cell proliferation and migration [117] (Fig. 4). While it was previously mentioned that zearalenone inhibits CRC growth in vivo by increasing the abundance of short-chain fatty acids-producing intestinal microbiota [99], another study found that zearalenone enhances GPER expression in colon cancer cell lines and activates the ERK1/2 and Hippo-YAP signaling pathways, thereby promoting cell proliferation [118]. The discrepancy between in vivo and in vitro findings regarding zearalenone’s effects might lead to seemingly contradictory conclusions. However, the consistent finding is GPER’s role in promoting CRC growth.

Collectively, the variability in GPER expression across different CRC cell lines indicates that GPER levels do not consistently correlate with tumorigenic or metastatic potential. High GPER expression has been specifically associated with a poorer prognosis in advanced female CRC patients. In CRC cells that have lost ERβ expression or contain *KRAS* MT, E2 interacts with GPER to inhibit cancer progression under normoxic conditions. However, this interaction initiates metabolic reprogramming and shifts towards promoting tumor growth under conditions of hypoxia and nutrient deficiency. E2-stimulated GPER activation can lead to centrosome amplification, increased STS activity, upregulated oncogene expression, and the activation of lipid metabolism pathways, ultimately promoting cell proliferation and metastasis (Fig. 4; Table 1).

**Table 1:** Potential mechanisms by which E2 signaling inhibits or promotes CRC progression

Model organism	Findings	Ref.
**Enhancing DNA mismatch repair and regulating epigenetic events**		
HT29, SW480 and LoVo cells	E2 enhances DNA mismatch repair and tumor suppression by inducing ERβ-mediated *MLH1* expression	[45]
OVX_CDX mice	E2 also enhanced the sensitivity of CRC cells to 5-FU and inhibits tumor proliferation in vivo and in vitro	
Human CRC tissue	Germline *ESR2*-CA genotype and resulting ERβ expression, as well as estrogen activation can influence MMR status in human CRC	[46]
DLD1 and Lovo cells	miR-34a strongly influences the expression of clock (*per2*) and clock controlled genes. E2 inhibits the migration and proliferation of DLD1 cells independently of miR-34a-mediated actions	[49]
COLO205 and SW480 cells	E2 regulates the expression of miRNAs and MMR genes through ERβ in CRC cells, thus exerting anticancer effects	[50]
Human CRC tissue		
HFD ERβ_KO mice	ERβ is a regulator of *bmal1* and *Npas2*, and E2 can regulate HFD-induced alterations in core clock genes *via* ERβ, thereby inhibiting colon cell proliferation	[58]
DLD1 and SW620 cells	E2 significantly inhibits the proliferation of CRC cell lines by activating the *CB1*-promoting region located in exon 1 of the *CNR1* gene, inducing the binding of ERβ to the *CNR1*-promoting region, and increasing the expression of the CB1	[60]
DLD1, HT29 and SW620 cells	E2 exerts antiproliferative properties by upregulating *CB1* gene expression in CRC cells	[61]
**Initiating cell cycle arrest and inducing apoptosis**		
SW480 and SW620 cells	E2-alone or combined with progesterone promotes cell cycle arrest in sub-G1 phase and apoptosis by stimulating the expression of ERβ and inhibiting ERα-regulated oncogenic pathways	[62]
male-AOM mice		
HT29, SW480 and SW620 cells	E2 monotherapy or in combination with 5-FU therapy has the potential to arrest the cell cycle progression at the sub-G1 phase and induce apoptosis in both female and male CRC cells, with the smallest effect in SW620 metastatic cells	[63]
Human CRC tissue	ERα proteins increased, whilst ERβ declined markedly in malignant specimens	[64]
SW480 and HT29 cells	E2 monotherapy induces cell cycle arrest and apoptosis in SW480 and HT29 cells, and pretreatment with ERα-blockers potentiates the effects of E2, whereas ERβ-blockers inhibits the E2 anti-cancer actions	
LoVo cells	E2 treatment reduces the expression of cell cycle regulatory proteins cyclin A, cyclin D1, cyclin E, and PCNA in a time-dependent manner, thereby inhibiting LoVo cell proliferation	[65]
COLO205 cells	E2 treatment reduces the expression of c-myb and its transcriptional target Bcl-2, thereby promoting COLO-205 cell apoptosis	[66]
**Inhibiting cell migration**		
DLD1 cells	E2 alone or in combination with tamoxifen inhibits DLD1 cells *via*bility and migration, promotes apoptosis, and decreases survivin mRNA and protein expression levels in a dose- and time-dependent manner	[67]
LoVo cells	E2 treatment significantly inhibits LoVo cell migration by downregulating the expression of cell migration-associated factor *via* activating E2/ERs-p38a MAPK and p53 signaling pathways	[65, 71]
LoVo cells	E2 inhibited the activities of uPA, MMP-9, and COX-2 by suppressing the JNK1/2, Akt and ERK1/2 pathways, therby inhibiting prostaglandin E2-induced LoVo cell migration.	[72, 73]
Apc (*Min/+* _OVX) mice	E2 prevent intestinal tumorigenesis and ameliorate enterocyte migration and intercellular adhesion in the *Apc*^*Min/+*^ mouse model of CRC	[75]
Colon CSCs	E2 has no effect on the *via*bility and proliferation of CSCs, but inhibits the exocytosis and exosome biogenesis, as well as reduces the affinity of CSCs for endothelial cells on the surface of the vascular lumen, leading to CSCs migration	[76]
**Stimulating ERβ expression and reducing inflammatory response**		
YAMC and YAMC-*Ras* cells	E2 inhibits nonmalignant YAMC cells growth and induces apoptosis, but has no effect on YAMC-*Ras* cells	[77]
OVX_ERβ_KO mice	E2 treatment inhibits the formation of ACFs in mouse colonic epithelium and shows higher apoptotic activity, which greatly reduces the occurrence of colonic preneoplastic lesions	
OVX_ERβ_KO AOM/DSS mice	Administration of E2 treatment after the onset of DNA damage reduces colon tumor formation. When colon epithelial cells progressed from a non-malignant to a cancerous state, decreased ERβ expression accompanied by upregulated ERα expression, and the presence of E2 did not affect the reduction in ERβ expression	[78]
DLD1 cells	E2 induces short-term translation and late transcriptional enhancement of ERβ mRNA in DLD1 cells through activation of p38/MAPK signaling.	[80]
OVX_ERβ_KO mice	E2 has different protective effects against chemical carcinogen-induced acute colitis and colon tumor formation in the presence or absence of ERβ	[81]
OVX_AOM/DSS mice	The protective effect of E2 against OVX_AOM/ DSS-induced colitis and carcinogenesis may involve in decreasing the levels of ERα but enhancing the expression of ERβ	[79]
	E2 inhibits the NF-κB pathway, enhances the expression of Nrf2 and Nrf2-associated antioxidant enzyme genes, and significantly increases the expression of NLRP3 inflammasome, thereby reducing the histological severity of colitis and preventing colorectal carcinogenesis	[82]
Nrf2_KO AOM/DSS mice	In the absence of Nrf2, E2 significantly inhibits the development of distal colon tumors through ERβ-related pathways.	[83]
CCD 841 CoN	E2 may inhibit inflammation in CCD841CoN cells by down-regulating the expression of inflammatory factors NF-κB and COX-2 as well as inducing the expression of the antioxidant enzymes HO-1 and NQO-1. And E2 treatment induced the expression of ERβ but had no effect on ERα	[84]
DLD1 cells	E2 stimulation upregulates neuroglobin expression in DLD1 cells. Neuroglobin serves as an oxidative stress sensor and facilitates E2-induced apoptosis in the absence of oxidative stress. However, under high oxidative stress conditions, neuroglobulin upregulation blocked the pro-apoptotic effect of E2	[85]
OVX_AOM/DSS mice	E2 promotes intestinal tumor development in the context of inflammatory injury. And ERα mediates a worsening of inflammation, whereas ERβ mediates pro-tumorigenic effects unrelated to inflammation	[86]
**Modulating immunosuppressive microenvironment**		
Nrf2_KO AOM/DSS mice	E2 treatment significantly reduces PD-L1, iNOS and COX-2 expression in Nrf2_KO AOM/DSS mice	[87]
MC38 cells	Different concentrations of E2 does not affect cell proliferation and apoptosis, nor does it affect the expression of PD-L1 in MC38 cells	[89]
OVX_MC38 tumor model mice	E2 may indirectly inhibit MC38 tumor growth by regulating the TIME through MC38-EVs	
OVX_MC38 tumor model mice	E2 is a facilitator of the pro-metastatic immune microenvironment in CRC liver lesions	[90]
MC38 tumor model mice	E2 inhibits MC38 tumor growth by reducing PD-L1 expression and regulating tumor-associated cell populations	[91]
OVX_MC38 tumor model mice	Lack of estrogen induced the formation of more tumor-promoting macrophages and accelerated tumor growth in HFD OVX_mice	[92]
HFD ERβ_KO mice	E2 inhibits HFD-induced F4/80^+^ macrophage infiltration and colon epithelial cell proliferation in male mice *via* ERβ, while reducing body weight and improving colon metabolic profile *via* ERα	[58]
**Regulating the gut microbiome composition and diversity**		
MC38 tumor model mice	E2 pre-treatment prior to anti-PD-L1 therapy increases the abundance of commensal bacteria while decreases the opportunistic pathogens, thereby contributing to anti-tumor therapy	[95]
AOM/DSS mice	E2 increases gut microbial diversity and the ratio of intestinal commensal bacteria to opportunistic pathogens, as well as decreases *Firmicutes*/*Bacteroidetes* ratios, thereby reducing the risk of CRC	[96]
*Apc*^Min/+^ and AOM/DSS mice	E2 enhances *C. maltaromaticum* attachment and intestinal colonization by up-regulating colonic SLC3A2 expression. *C. maltaromaticum* together with other microbiome to activate the vitamin D receptor signaling to inhibit CRC	[97]
NCM460 cells		
AOM/DSS mice	Intestinal ERβ contributes to a more favorable microbiome and assists E2 in delaying the progression of CAC	[98]
AOM/DSS mice	The estrogenic mycotoxin zearalenone increases SCFAs-producing intestinal microbiome with good inhibitory effects on CRC	[99]
**GPER**-**mediated non**-**genomic signaling of E2**		
HT29, HCT116 and DLD1 cells	E2, acting through GPER, inhibits ERβ-negative CRC cell migration and proliferation under normoxic conditions but produces opposite tumor-promoting effects under hypoxic conditions	[38]
Human CRC tissue	High GPER expression is significantly associated with shorter survival in female CRC patients at stage III-IV, but not in male CRC patients at any stage	
3D organoid model of *KRAS* MT and *KRAS* WT CRC cells	E2 inhibits *KRAS* WT cells growth but has no effect on *KRAS* MT cells. *KRAS* MT cells resist the protective effect of E2 through GPER1 under nutrient-depleted conditions	[35]
SW480 and COLO205 cells	E2 does not significantly affect GPER expression in SW480 and COLO205 cells	[114]
HCT116 and CCD841CoN cells	GPER1 activated by high concentrations of E2 induces excessive centrosome amplification in CRC cells, leading to transient production of multipolar mitotic spindles, alignment-defective and lagging chromosomes	[115]
Col205, Caco2, HCT116 and HT29 cells	Elevated STS activity in CRC promotes the pathway of E_1_S hydrolysis and subsequent E2 synthesis. STS-activated E2 stimulates GPER and promotes CTGF expression, thereby driving cell proliferation	[39, 116]
CDX		
Human CRC tissue		
LoVo cells	E2 induces fatty acid synthase expression in ERs-negative CRC cells and CAFs through the GPER-mediated EGFR/ERK/c-Fos/AP1 transduction signaling, thereby stimulating cancer cell proliferation and migration	[117]

*Note ACF*s aberrant crypt focis, *CAC* colitis-associated cancer, *CAF*s cancer-associated fibroblasts, *CB1* cannabinoid receptor 1, *CSC*s cancer stem cells, *CDX* cell line-derived xenograft, *CRC* colorectal cancer, *CTGF* connective tissue growth factor, *E*_*1*_*S* estrone sulfate, *E2* 17β-estradiol, *ERs* estrogen receptors, *EVs* extracellular vesicles, *F/B Firmicutes*/*Bacteroidetes*, *GPER* G protein-coupled estrogen receptor, *HFD* high-fat diet, *HRT* Hormone replacement therapy, *IGF*-1 insulin-like growth factor 1, *KO* knockout, *MMPs* matrix metalloproteinases, *MMR* mismatch repair gene, *MT* mutant, *OVX* ovariectomized, *PAs* plasminogen activators, *PD-L1* programmed death-ligand 1, *SCFAs* short chain fatty acids, *STS* steroid sulfatase, *TIME* tumor immune microenvironment, *TME* tumor microenvironment, *WT* wild-type, *YAMC* young adult mouse colonocytes

## Conclusion and future perspectives

In conclusion, at the cellular level, E2 inhibits CRC cell proliferation, invasion, and migration through various mechanisms, including enhancing DNA mismatch repair, regulating miRNA and core clock gene expression, initiating cell cycle arrest and apoptosis, and inhibiting cell migration factor activity. Notably, the stimulation of ERβ expression is a key mechanism through which E2 exerts its protective effects in CRC. E2 mitigates inflammatory responses and oxidative stress damage through ERβ, thereby impeding early CRC progression. Moreover, E2 signaling reverses the immunosuppressive microenvironment by decreasing PD-L1 expression and regulating immune cell infiltration and tumor-associated cell populations. Additionally, E2 can alter the gut microbiome composition to reduce CRC risk by increasing the ratio of commensal bacteria to opportunistic pathogens, adjusting the F/B ratio, promoting the colonization of probiotics, and increasing the abundance of SCFAs-producing bacteria. In turn, the gut microbiome can influence E2 levels through multiple pathways. Conversely, in ERβ-negative CRC, E2 may inhibit cancer progression through GPER under normoxic conditions but exhibit pro-tumorigenic effects under hypoxic and nutrient-deficient conditions. E2-stimulated GPER activation in CRC cells can lead to centrosome amplification, increased STS activity, upregulated oncogene expression, and the activation of lipid metabolism pathways, ultimately promoting cell proliferation and metastasis. Moreover, a high-fat diet and obesity may pose additional risk factors for CRC.

Current research has primarily focused on CRC cells and animal models, which can originate from humans or mice of varying sexes, ages, and tumor stages. Animal models also vary between colitis-induced cancers and xenograft tumors, resulting in differences in ERs in CRC cells or tissues. However, some studies have not thoroughly examined ERs expression or clarified whether E2 affects CRC directly or indirectly. Furthermore, the concentration and duration of exposure to E2 vary in the literature. These seem to partially explain the seemingly contradictory roles of E2 in CRC. Therefore, future studies should refine CRC models, accurately assess ER expression, standardize E2 concentration and exposure time, and differentiate between direct and indirect pathways of E2 action. Overall, E2 signaling in CRC exhibits dual effects of both anticancer and carcinogenic properties through intricate transduction pathways, likely influenced by factors such as E2 concentration and exposure time, ERs type and expression levels, individual variations, and the TME (e.g., oxygen levels, nutritional status). E2 modulates genomic and non-genomic signaling *via* ERβ and GPER, respectively, predominantly suppressing early CRC lesions. Moreover, E2 can directly impact CRC cell proliferation, invasion, and migration, while also indirectly influencing CRC progression by modulating colonic inflammation, immunity, and the gut microbiome. These insights help elucidate sex-based disparities in CRC incidence and prognosis, as well as inform the evaluation of HRT in CRC patients, offering a scientific foundation for CRC prevention and treatment.

## Data Availability

No datasets were generated or analysed during the current study.

## Abbreviations

ACFs: Aberrant crypt focis
AF-1/2: Activation function-1/2
ASNS: Asparagine synthase
ATM: Ataxia telangiectasia mutated
CAC: Colitis-associated cancer
CAFs: Cancer-associated fibroblasts
CB1: Cannabinoid receptor 1
CSCs: Cancer stem cells
CDX: Cell line-derived xenograft
CRC: Colorectal cancer
CTGF: Connective tissue growth factor
C/O: Commensal bacteria/opportunistic pathogens
DBD: DNA binding domain
E_1_S: Estrone sulfate
E2: 17β-estradiol
ECM: Extracellular matrix
ERE: Estrogen-responsive element
ERs: Estrogen receptors
EVs: Extracellular vesicles
F/B: Firmicutes/Bacteroidetes
GPER: G protein-coupled estrogen receptor
HFD: High-fat diet
HRT: Hormone replacement therapy
IGF-1: Insulin-like growth factor 1
KO: Knockout
LBD: Ligand binding domain
MDSCs: Myeloid-derived suppressor cells
MMPs: Matrix metalloproteinases
MMR: Mismatch repair
MSI: Microsatellite instability
MT: Mutant
NTD: NH2-terminal domain
OVX: Ovariectomized
PAs: Plasminogen activators
PD-L1: Programmed death-ligand 1
SCFAs: Short chain fatty acids
STS: Steroid sulfatase
TAMs: Tumor-associated macrophages
TIME: Tumor immune microenvironment
TME: Tumor microenvironment
VEGFA: Vascular endothelial growth factor
WT: Wild-type
YAMC: Young adult mouse colonocytes

## References

[1] Siegel RL, Wagle NS, Cercek A, Smith RA, Jemal A. Colorectal cancer statistics, 2023. CA Cancer J Clin. 2023;73:233–54. 36856579 10.3322/caac.21772

[2] Patel SG, Karlitz JJ, Yen T, Lieu CH, Boland CR. The rising tide of early-onset colorectal cancer: a comprehensive review of epidemiology, clinical features, biology, risk factors, prevention, and early detection. Lancet Gastroenterol Hepatol. 2022;7:262–74. 35090605 10.1016/S2468-1253(21)00426-X

[3] Siegel RL, Giaquinto AN, Jemal A. Cancer statistics, 2024. CA Cancer J Clin. 2024;74:12–49. 38230766 10.3322/caac.21820

[4] Murphy N, Strickler HD, Stanczyk FZ, Xue X, Wassertheil-Smoller S, Rohan TE, Ho GY, Anderson GL, Potter JD, Gunter MJ. A prospective evaluation of endogenous sex hormone levels and colorectal Cancer risk in Postmenopausal Women. J Natl Cancer Inst. 2015;107:djv210. 26232761 10.1093/jnci/djv210 PMC5964717

[5] Razak S, Alam I, Afsar T, Abulmeaty MMA, Almajwal A, Jahan S. A Prospective Evaluation of Serum Vitamin D (1, 25(OH)(2) D(3)) and endogenous sex hormone levels in Colorectal Cancer patients. Front Oncol. 2019;9:468. 31214508 10.3389/fonc.2019.00468 PMC6558010

[6] Lin JH, Zhang SM, Rexrode KM, Manson JE, Chan AT, Wu K, Tworoger SS, Hankinson SE, Fuchs C, Gaziano JM, et al. Association between sex hormones and colorectal cancer risk in men and women. Clin Gastroenterol Hepatol. 2013;11:419–e424411. 23200979 10.1016/j.cgh.2012.11.012 PMC3594467

[7] Lavasani S, Chlebowski RT, Prentice RL, Kato I, Wactawski-Wende J, Johnson KC, Young A, Rodabough R, Hubbell FA, Mahinbakht A, Simon MS. Estrogen and colorectal cancer incidence and mortality. Cancer. 2015;121:3261–71. 26036212 10.1002/cncr.29464

[8] Mori N, Sawada N, Iwasaki M, Yamaji T, Goto A, Shimazu T, Inoue M, Murphy N, Gunter MJ, Tsugane S. Circulating sex hormone levels and colorectal cancer risk in Japanese postmenopausal women: the JPHC nested case-control study. Int J Cancer. 2019;145:1238–44. 31131883 10.1002/ijc.32431

[9] Mori N, Keski-Rahkonen P, Gicquiau A, Rinaldi S, Dimou N, Harlid S, Harbs J, Van Guelpen B, Aune D, Cross AJ, et al. Endogenous circulating sex hormone concentrations and Colon cancer risk in Postmenopausal women: a prospective study and Meta-analysis. JNCI Cancer Spectr. 2021;5:pkab084. 34805742 10.1093/jncics/pkab084 PMC8598284

[10] Yang W, Giovannucci EL, Hankinson SE, Chan AT, Ma Y, Wu K, Fuchs CS, Lee IM, Sesso HD, Lin JH, Zhang X. Endogenous sex hormones and colorectal cancer survival among men and women. Int J Cancer. 2020;147:920–30. 31863463 10.1002/ijc.32844 PMC7895324

[11] Chlebowski RT, Wactawski-Wende J, Ritenbaugh C, Hubbell FA, Ascensao J, Rebecca J, Rodabough, Rosenberg CA, Taylor VM, Harris R, Chen C, et al. Estrogen plus Progestin and Colorectal Cancer in Postmenopausal Women. N Engl J Med. 2004;350:991–1004. 14999111 10.1056/NEJMoa032071

[12] Tian Y, Lin Y, Qu C, Arndt V, Baurley JW, Berndt SI, Bien SA, Bishop DT, Brenner H, Buchanan DD, et al. Genetic risk impacts the association of menopausal hormone therapy with colorectal cancer risk. Br J Cancer. 2024;130:1687–96. 38561434 10.1038/s41416-024-02638-2 PMC11091089

[13] Amitay EL, Carr PR, Jansen L, Alwers E, Roth W, Herpel E, Kloor M, Bläker H, Chang-Claude J, Brenner H, Hoffmeister M. Postmenopausal hormone replacement therapy and colorectal cancer risk by molecular subtypes and pathways. Int J Cancer. 2020;147:1018–26. 31943160 10.1002/ijc.32868

[14] Thomas MP, Potter BV. The structural biology of oestrogen metabolism. J Steroid Biochem Mol Biol. 2013;137:27–49. 23291110 10.1016/j.jsbmb.2012.12.014 PMC3866684

[15] Chauvin S, Cohen-Tannoudji J, Guigon CJ. Estradiol Signaling at the heart of Folliculogenesis: its potential deregulation in human ovarian pathologies. Int J Mol Sci 2022, 23. 10.3390/ijms23010512 PMC8745567 35008938

[16] Acconcia F, Fiocchetti M, Marino M. Xenoestrogen regulation of ERalpha/ERbeta balance in hormone-associated cancers. Mol Cell Endocrinol. 2017;457:3–12. 27816767 10.1016/j.mce.2016.10.033

[17] Jia M, Dahlman-Wright K, Gustafsson JA. Estrogen receptor alpha and beta in health and disease. Best Pract Res Clin Endocrinol Metab. 2015;29:557–68. 26303083 10.1016/j.beem.2015.04.008

[18] Khan MZI, Uzair M, Nazli A, Chen JZ. An overview on estrogen receptors signaling and its ligands in breast cancer. Eur J Med Chem. 2022;241:114658. 35964426 10.1016/j.ejmech.2022.114658

[19] Rawluszko-Wieczorek AA, Romanowska K, Nowicki M. Chromatin modifiers - coordinators of estrogen action. Biomed Pharmacother 2022, 153. 10.1016/j.biopha.2022.113548 36076614

[20] Cerutti C, Shi J-R, Vanacker J-M. Multifaceted Transcriptional Network of Estrogen-Related Receptor Alpha in Health and Disease. Int J Mol Sci 2023, 24. 10.3390/ijms24054265 PMC10002233 36901694

[21] Arao Y, Korach KS. The physiological role of estrogen receptor functional domains. Essays Biochem. 2021;65:867–75. 34028522 10.1042/EBC20200167 PMC8611119

[22] Mauvais-Jarvis F, Clegg DJ, Hevener AL. The role of estrogens in control of energy balance and glucose homeostasis. Endocr Rev. 2013;34:309–38. 23460719 10.1210/er.2012-1055 PMC3660717

[23] Liu YW, Gao H, Marstrand TT, Ström A, Valen E, Sandelin A, Gustafsson JÅ, Dahlman-Wright K. The genome landscape of ERα- and ERβ-binding DNA regionsvol 105, pg 2604, (2008). *Proceedings of the National Academy of Sciences of the United States of America* 2019, 116:1460–1461. 10.1073/pnas.0712085105 PMC2268183 18272478

[24] Song DD, He H, Indukuri R, Huang ZQ, Stepanauskaite L, Sinha I, Haldosen LA, Zhao CY, Williams C. ERα and ERβ homodimers in the same Cellular Context regulate distinct transcriptomes and functions. Front Endocrinol 2022, 13. 10.3389/fendo.2022.930227 PMC9299245 35872983

[25] Mahboobifard F, Pourgholami MH, Jorjani M, Dargahi L, Amiri M, Sadeghi S, Tehrani FR. Estrogen as a key regulator of energy homeostasis and metabolic health. Biomed Pharmacother. 2022;156:113808. 36252357 10.1016/j.biopha.2022.113808

[26] Topi G, Satapathy SR, Dash P, Fred Mehrabi S, Ehrnström R, Olsson R, Lydrup ML, Sjölander A. Tumour-suppressive effect of oestrogen receptor β in colorectal cancer patients, colon cancer cells, and a zebrafish model. J Pathol. 2020;251:297–309. 32333795 10.1002/path.5453

[27] Topi G, Ghatak S, Satapathy SR, Ehrnström R, Lydrup M-L, Sjölander A. Combined estrogen alpha and Beta receptor expression has a prognostic significance for Colorectal Cancer patients. Front Med 2022, 9. 10.3389/fmed.2022.739620 PMC8963951 35360718

[28] Topi G, Satapathy SR, Ghatak S, Hellman K, Ek F, Olsson R, Ehrnström R, Lydrup M-L, Sjölander A. High oestrogen receptor alpha expression correlates with adverse prognosis and promotes metastasis in colorectal cancer. Cell Communication Signal 2024, 22. 10.1186/s12964-024-01582-1 PMC10979551 38549115

[29] Liu T, Zhao M, Peng L, Chen J, Xing P, Gao P, Chen L, Qiao X, Wang Z, Di J et al. WFDC3 inhibits tumor metastasis by promoting the ERβ-mediated transcriptional repression of TGFBR1 in colorectal cancer. Cell Death Dis 2023, 14. 10.1038/s41419-023-05956-0 PMC10345115 37443102

[30] Giroux V, Bernatchez G, Carrier JC. Chemopreventive Effect of ERβ-Selective Agonist on Intestinal Tumorigenesis in mice. Mol Carcinog. 2011;50:359–69. 21480389 10.1002/mc.20719

[31] Arterburn JB, Prossnitz ER. G protein-coupled estrogen receptor GPER: molecular pharmacology and therapeutic applications. Annu Rev Pharmacol Toxicol. 2023;63:295–320. 36662583 10.1146/annurev-pharmtox-031122-121944 PMC10153636

[32] Sharma G, Mauvais-Jarvis F, Prossnitz ER. Roles of G protein-coupled estrogen receptor GPER in metabolic regulation. J Steroid Biochem Mol Biol. 2018;176:31–7. 28223150 10.1016/j.jsbmb.2017.02.012 PMC5563497

[33] Prossnitz ER, Barton M. The G protein-coupled oestrogen receptor GPER in health and disease: an update. Nat Reviews Endocrinol. 2023;19:407–24. 10.1038/s41574-023-00822-7 PMC10187525 37193881

[34] Hall KA, Filardo EJ. The G protein-coupled estrogen receptor (GPER): a critical therapeutic target for Cancer. Cells 2023, 12. 10.3390/cells12202460 PMC10605794 37887304

[35] Lu L, Zhang Q, Shen X, Zhen P, Marin A, Garcia-Milian R, Roper J, Khan SA, Johnson CH. Asparagine synthetase and G-protein coupled estrogen receptor are critical responders to nutrient supply in KRAS mutant colorectal cancer. *bioRxiv* 2023. 10.1002/ijc.35104 PMC11537827 39039782

[36] Liu Q, Chen Z, Jiang G, Zhou Y, Yang X, Huang H, Liu H, Du J, Wang H. Epigenetic down regulation of G protein-coupled estrogen receptor (GPER) functions as a tumor suppressor in colorectal cancer. Mol Cancer. 2017;16:87. 28476123 10.1186/s12943-017-0654-3 PMC5418684

[37] Abancens M, Harvey BJ, McBryan J. GPER Agonist G1 prevents wnt-Induced JUN Upregulation in HT29 Colorectal Cancer cells. Int J Mol Sci 2022, 23. 10.3390/ijms232012581 PMC9603962 36293473

[38] Bustos V, Nolan ÁM, Nijhuis A, Harvey H, Parker A, Poulsom R, McBryan J, Thomas W, Silver A, Harvey BJ. GPER mediates differential effects of estrogen on colon cancer cell proliferation and migration under normoxic and hypoxic conditions. Oncotarget. 2017;8:84258–75. 29137421 10.18632/oncotarget.20653 PMC5663593

[39] Gilligan LC, Rahman HP, Hewitt AM, Sitch AJ, Gondal A, Arvaniti A, Taylor AE, Read ML, Morton DG, Foster PA. Estrogen activation by Steroid Sulfatase increases Colorectal Cancer Proliferation via GPER. J Clin Endocrinol Metab. 2017;102:4435–47. 28945888 10.1210/jc.2016-3716 PMC5718700

[40] Jacenik D, Beswick EJ, Krajewska WM, Prossnitz ER. G protein-coupled estrogen receptor in colon function, immune regulation and carcinogenesis. World J Gastroenterol. 2019;25:4092–104. 31435166 10.3748/wjg.v25.i30.4092 PMC6700692

[41] Taieb J, Svrcek M, Cohen R, Basile D, Tougeron D, Phelip JM. Deficient mismatch repair/microsatellite unstable colorectal cancer: diagnosis, prognosis and treatment. Eur J Cancer. 2022;175:136–57. 36115290 10.1016/j.ejca.2022.07.020

[42] Lizardo DY, Kuang C, Hao S, Yu J, Huang Y, Zhang L. Immunotherapy efficacy on mismatch repair-deficient colorectal cancer: From bench to bedside. *Biochimica et Biophysica Acta (BBA) - Reviews on Cancer* 2020, 1874. 10.1016/j.bbcan.2020.188447 PMC7886024 33035640

[43] Jin P, Lu XJ, Sheng JQ, Fu L, Meng XM, Wang X, Shi TP, Li SR, Rao J. Estrogen stimulates the expression of mismatch repair gene hMLH1 in colonic epithelial cells. Cancer Prev Res (Phila). 2010;3:910–6. 20663978 10.1158/1940-6207.CAPR-09-0228

[44] Sengodan SK, Hu X, Peddibhotla V, Balamurugan K, Mitrophanov AY, McKennett L, Kharat SS, Sanawar R, Singh VK, Albaugh ME et al. Mismatch repair protein MLH1 suppresses replicative stress in BRCA2 deficient breast tumors. J Clin Invest 2024. 10.1172/JCI173718 PMC10977984 38271119

[45] Lu J, Jin P, Gao W, Wang D, Sheng J. Estrogen enhances mismatch repair by induction of MLH1 expression via estrogen receptor-β. Oncotarget. 2017;8:38767–79. 28404976 10.18632/oncotarget.16351 PMC5503570

[46] Honma N, Arai T, Matsuda Y, Fukunaga Y, Muramatsu M, Ikeda S, Akishima-Fukasawa Y, Yamamoto N, Kawachi H, Ishikawa Y et al. Estrogen receptor-beta gene cytosine-adenine (ESR2-CA) repeat polymorphism in postmenopausal Colon cancer. Int J Mol Sci 2023, 24. 10.3390/ijms24054502 PMC10003297 36901930

[47] Wang S, Sun Z, Lei Z, Zhang HT. RNA-binding proteins and cancer metastasis. Semin Cancer Biol. 2022;86:748–68. 35339667 10.1016/j.semcancer.2022.03.018

[48] Smolarz B, Durczynski A, Romanowicz H, Szyllo K, Hogendorf P. miRNAs in Cancer (Review of Literature). Int J Mol Sci 2022, 23. 10.3390/ijms23052805 PMC8910953 35269947

[49] Moravcik R, Olejarova S, Zlacka J, Herichova I. Effect of miR-34a on the expression of clock and clock-controlled genes in DLD1 and Lovo human cancer cells with different backgrounds with respect to p53 functionality and 17beta-estradiol-mediated regulation. PLoS ONE. 2023;18:e0292880. 37831728 10.1371/journal.pone.0292880 PMC10575541

[50] He YQ, Sheng JQ, Ling XL, Fu L, Jin P, Yen L, Rao J. Estradiol regulates miR-135b and mismatch repair gene expressions via estrogen receptor-beta in colorectal cells. Exp Mol Med. 2012;44:723–32. 23143558 10.3858/emm.2012.44.12.079 PMC3538979

[51] Vymetalkova V, Pardini B, Rosa F, Di Gaetano C, Novotny J, Levy M, Buchler T, Slyskova J, Vodickova L, Naccarati A, Vodicka P. Variations in mismatch repair genes and colorectal cancer risk and clinical outcome. Mutagenesis. 2014;29:259–65. 24755277 10.1093/mutage/geu014

[52] Ye L, Jiang T, Shao HZ, Zhong L, Wang ZW, Liu Y, Tang HM, Qin BY, Zhang XQ, Fan JW. miR-1290 is a biomarker in DNA-Mismatch-repair-deficient Colon cancer and promotes resistance to 5-Fluorouracil by directly targeting hMSH2. Mol Therapy-Nucleic Acids. 2017;7:453–64. 10.1016/j.omtn.2017.05.006 PMC5443909 28624221

[53] Liang GF, Zhu YL, Ali DJ, Tian T, Xu HT, Si K, Sun B, Chen BA, Xiao ZD. Engineered exosomes for targeted co-delivery of miR-21 inhibitor and chemotherapeutics to reverse drug resistance in colon cancer. J Nanobiotechnol 2020, 18. 10.1186/s12951-019-0563-2 PMC6950820 31918721

[54] Valeri N, Gasparini P, Braconi C, Paone A, Lovat F, Fabbri M, Sumani KM, Alder H, Amadori D, Patel T et al. MicroRNA-21 induces resistance to 5-fluorouracil by down-regulating human DNA MutS homolog 2 (hMSH2). *Proceedings of the National Academy of Sciences* 2010, 107:21098–21103. 10.1073/pnas.1015541107 PMC3000294 21078976

[55] Ruan W, Yuan XY, Eltzschig HK. Circadian rhythm as a therapeutic target. Nat Rev Drug Discovery. 2021;20:287–307. 33589815 10.1038/s41573-020-00109-w PMC8525418

[56] Zhou L, Zhang Z, Nice E, Huang CH, Zhang W, Tang Y. Circadian rhythms and cancers: the intrinsic links and therapeutic potentials. J Hematol Oncol 2022, 15. 10.1186/s13045-022-01238-y PMC8896306 35246220

[57] Levi FA, Okyar A, Hadadi E, Innominato PF, Ballesta A. Circadian regulation of drug responses: toward sex-specific and personalized chronotherapy. Annu Rev Pharmacol Toxicol. 2024;64:89–114. 37722720 10.1146/annurev-pharmtox-051920-095416

[58] Hases L, Archer A, Indukuri R, Birgersson M, Savva C, Korach-Andre M, Williams C. High-fat diet and estrogen impacts the colon and its transcriptome in a sex-dependent manner. Sci Rep. 2020;10:16160. 32999402 10.1038/s41598-020-73166-1 PMC7527340

[59] Olejarova S, Moravcik R, Herichova I. 2.4 GHz Electromagnetic Field influences the response of the circadian oscillator in the Colorectal Cancer Cell Line DLD1 to miR-34a-Mediated regulation. Int J Mol Sci 2022, 23. 10.3390/ijms232113210 PMC9656412 36361993

[60] Proto MC, Gazzerro P, Di Croce L, Santoro A, Malfitano AM, Pisanti S, Laezza C, Bifulco M. Interaction of endocannabinoid system and steroid hormones in the control of colon cancer cell growth. J Cell Physiol. 2012;227:250–8. 21412772 10.1002/jcp.22727

[61] Notarnicola M, Messa C, Orlando A, Bifulco M, Laezza C, Gazzerro P, Caruso MG. Estrogenic induction of cannabinoid CB1 receptor in human colon cancer cell lines. Scand J Gastroenterol. 2008;43:66–72. 18938775 10.1080/00365520701559011

[62] Mahbub AA, Aslam A, Elzubier ME, El-Boshy M, Abdelghany AH, Ahmad J, Idris S, Almaimani R, Alsaegh A, El-Readi MZ, et al. Enhanced anti-cancer effects of oestrogen and progesterone co-therapy against colorectal cancer in males. Front Endocrinol (Lausanne). 2022;13:941834. 36263327 10.3389/fendo.2022.941834 PMC9574067

[63] Mahbub AA. 17beta-estradiol enhances 5-Fluorouracil anti-cancer activities in Colon cancer cell lines. Med Sci (Basel). 2022;10:62. 36412903 10.3390/medsci10040062 PMC9680382

[64] Refaat B, Aslam A, Idris S, Almalki AH, Alkhaldi MY, Asiri HA, Almaimani RA, Mujalli A, Minshawi F, Alamri SA, et al. Profiling estrogen, progesterone, and androgen receptors in colorectal cancer in relation to gender, menopausal status, clinical stage, and tumour sidedness. Front Endocrinol (Lausanne). 2023;14:1187259. 37206439 10.3389/fendo.2023.1187259 PMC10190606

[65] Hsu HH, Liu CJ, Shen CY, Chen YJ, Chen LM, Kuo WH, Lin YM, Chen RJ, Tsai CH, Tsai FJ, Huang CY. p38alpha MAPK mediates 17beta-estradiol inhibition of MMP-2 and – 9 expression and cell migration in human lovo colon cancer cells. J Cell Physiol. 2012;227:3648–60. 22377968 10.1002/jcp.24072

[66] Wilkins HR, Doucet K, Duke V, Morra A, Johnson N. Estrogen prevents sustained COLO-205 human colon cancer cell growth by inducing apoptosis, decreasing c-myb protein, and decreasing transcription of the anti-apoptotic protein bcl-2. Tumour Biol. 2010;31:16–22. 20237898 10.1007/s13277-009-0003-2

[67] Ou QJ, Wu XJ, Peng JH, Zhang RX, Lu ZH, Jiang W, Zhang L, Pan ZZ, Wan DS, Fang YJ. Endocrine therapy inhibits proliferation and migration, promotes apoptosis and suppresses survivin protein expression in colorectal cancer cells. Mol Med Rep. 2017;16:5769–78. 28849238 10.3892/mmr.2017.7375 PMC5865723

[68] Jiang Y, Zhang H, Wang J, Liu Y, Luo T, Hua H. Targeting extracellular matrix stiffness and mechanotransducers to improve cancer therapy. J Hematol Oncol. 2022;15:34. 35331296 10.1186/s13045-022-01252-0 PMC8943941

[69] de Almeida LGN, Thode H, Eslambolchi Y, Chopra S, Young D, Gill S, Devel L, Dufour A. Matrix metalloproteinases: from Molecular mechanisms to Physiology, Pathophysiology, and Pharmacology. Pharmacol Rev. 2022;74:712–68. 35738680 10.1124/pharmrev.121.000349

[70] Ismail AA, Shaker BT, Bajou K. The plasminogen-activator plasmin system in physiological and pathophysiological angiogenesis. Int J Mol Sci 2021, 23. 10.3390/ijms23010337 PMC8745544 35008762

[71] Hsu HH, Kuo WW, Ju DT, Yeh YL, Tu CC, Tsai YL, Shen CY, Chang SH, Chung LC, Huang CY. Estradiol agonists inhibit human LoVo colorectal-cancer cell proliferation and migration through p53. World J Gastroenterol. 2014;20:16665–73. 25469035 10.3748/wjg.v20.i44.16665 PMC4248210

[72] Lai TY, Chen LM, Lin JY, Tzang BS, Lin JA, Tsai CH, Lin YM, Huang CY, Liu CJ, Hsu HH. 17beta-estradiol inhibits prostaglandin E2-induced COX-2 expressions and cell migration by suppressing Akt and ERK1/2 signaling pathways in human LoVo colon cancer cells. Mol Cell Biochem. 2010;342:63–70. 20446020 10.1007/s11010-010-0469-7

[73] HH H, WS H, YM L, WW K, LM C, WK C, JM H, FJ T, CJ L, CY H. JNK suppression is essential for 17b-Estradiol inhibits prostaglandin E2-Induced uPA and MMP9 expressions and cell migration in human LoVo colon cancer cells. J Biomed Sci 2011, 18. 10.1186/1423-0127-18-61 PMC3179949 21859479

[74] Beumer J, Puschhof J, Yengej FY, Zhao L, Martinez-Silgado A, Blotenburg M, Begthel H, Boot C, van Oudenaarden A, Chen YG, Clevers H. BMP gradient along the intestinal villus axis controls zonated enterocyte and goblet cell states. Cell Rep. 2022;38:110438. 35235783 10.1016/j.celrep.2022.110438

[75] Javid SH, Moran AE, Carothers AM, Redston M, Bertagnolli MM. Modulation of tumor formation and intestinal cell migration by estrogens in the apc(Min/+) mouse model of colorectal cancer. Carcinogenesis. 2005;26:587–95. 15579483 10.1093/carcin/bgh346

[76] Zamani ARN, Avci CB, Ahmadi M, Pouyafar A, Bagheri HS, Fathi F, Heidarzadeh M, Rezaie J, Mirhosseini Y, Saberianpour S, et al. Estradiol modulated colorectal cancer stem cells bioactivity and interaction with endothelial cells. Life Sci. 2020;257:118078. 32663577 10.1016/j.lfs.2020.118078

[77] Weige CC, Allred KF, Allred CD. Estradiol alters cell growth in nonmalignant colonocytes and reduces the formation of preneoplastic lesions in the colon. Cancer Res. 2009;69:9118–24. 19903848 10.1158/0008-5472.CAN-09-2348

[78] Armstrong CM, Billimek AR, Allred KF, Sturino JM, Weeks BR, Allred CD. A novel shift in estrogen receptor expression occurs as estradiol suppresses inflammation-associated colon tumor formation. Endocr Relat Cancer. 2013;20:515–25. 23702470 10.1530/ERC-12-0308

[79] Song CH, Kim N, Lee SM, Nam RH, Choi SI, Kang SR, Shin E, Lee DH, Lee HN, Surh YJ. Effects of 17beta-estradiol on colorectal cancer development after azoxymethane/dextran sulfate sodium treatment of ovariectomized mice. Biochem Pharmacol. 2019;164:139–51. 30981879 10.1016/j.bcp.2019.04.011

[80] Caiazza F, Galluzzo P, Lorenzetti S, Marino M. 17Beta-estradiol induces ERbeta up-regulation via p38/MAPK activation in colon cancer cells. Biochem Biophys Res Commun. 2007;359:102–7. 17524358 10.1016/j.bbrc.2007.05.059

[81] Armstrong CM, Allred KF, Weeks BR, Chapkin RS, Allred CD. Estradiol has Differential effects on Acute Colonic inflammation in the Presence and absence of estrogen receptor beta expression. Dig Dis Sci. 2017;62:1977–84. 28573506 10.1007/s10620-017-4631-x PMC5751962

[82] Son HJ, Sohn SH, Kim N, Lee HN, Lee SM, Nam RH, Park JH, Song CH, Shin E, Na HY, et al. Effect of Estradiol in an Azoxymethane/Dextran sulfate sodium-treated mouse model of Colorectal Cancer: implication for sex difference in Colorectal Cancer Development. Cancer Res Treat. 2019;51:632–48. 30064198 10.4143/crt.2018.060 PMC6473282

[83] Song CH, Kim N, Hee Nam R. In Choi S, Hee Son J, Eun Yu J, Shin E, Lee HN, Kim DH, Surh YJ: 17beta-Estradiol strongly inhibits azoxymethane/dextran sulfate sodium-induced colorectal cancer development in Nrf2 knockout male mice. *Biochem Pharmacol* 2020, 182:114279. 10.1016/j.bcp.2020.114279 33068552

[84] Son HJ, Kim N, Song CH, Lee SM, Lee HN, Surh YJ. 17beta-Estradiol reduces inflammation and modulates antioxidant enzymes in colonic epithelial cells. Korean J Intern Med. 2020;35:310–9. 30336658 10.3904/kjim.2018.098 PMC7061017

[85] Fiocchetti M, Camilli G, Acconcia F, Leone S, Ascenzi P, Marino M. ERbeta-dependent neuroglobin up-regulation impairs 17beta-estradiol-induced apoptosis in DLD-1 colon cancer cells upon oxidative stress injury. J Steroid Biochem Mol Biol. 2015;149:128–37. 25683270 10.1016/j.jsbmb.2015.02.005

[86] Heijmans J, Wielenga MC, Rosekrans SL, van Lidth de Jeude JF, Roelofs J, Groothuis P, Ederveen A, de Jonge-Muller ES, Biemond I, Hardwick JC, et al. Oestrogens promote tumorigenesis in a mouse model for colitis-associated cancer. Gut. 2014;63:310–6. 23408349 10.1136/gutjnl-2012-304216

[87] Kang C, Song CH, Kim N, Nam RH, Choi SI, Yu JE, Nho H, Choi JA, Kim JW, Na HY, et al. The enhanced inhibitory effect of Estrogen on PD-L1 expression following Nrf2 Deficiency in the AOM/DSS Model of Colitis-Associated Cancer. Front Oncol. 2021;11:679324. 34307147 10.3389/fonc.2021.679324 PMC8297827

[88] Dammeijer F, van Gulijk M, Mulder EE, Lukkes M, Klaase L, van den Bosch T, van Nimwegen M, Lau SP, Latupeirissa K, Schetters S, et al. The PD-1/PD-L1-Checkpoint restrains T cell immunity in Tumor-Draining Lymph Nodes. Cancer Cell. 2020;38:685–e700688. 33007259 10.1016/j.ccell.2020.09.001

[89] Jiang L, Fei H, Yang A, Zhu J, Sun J, Liu X, Xu W, Yang J, Zhang S. Estrogen inhibits the growth of colon cancer in mice through reversing extracellular vesicle-mediated immunosuppressive tumor microenvironment. Cancer Lett. 2021;520:332–43. 34391809 10.1016/j.canlet.2021.08.011

[90] Milette S, Hashimoto M, Perrino S, Qi S, Chen M, Ham B, Wang N, Istomine R, Lowy AM, Piccirillo CA, Brodt P. Sexual dimorphism and the role of estrogen in the immune microenvironment of liver metastases. Nat Commun. 2019;10:5745. 31848339 10.1038/s41467-019-13571-x PMC6917725

[91] Song CH, Kim N, Nam RH, Choi SI, Jang JY, Kim JW, Na HY, Lee HN. Combination treatment with 17beta-estradiol and anti-PD-L1 suppresses MC38 tumor growth by reducing PD-L1 expression and enhancing M1 macrophage population in MC38 colon tumor model. Cancer Lett. 2022;543:215780. 35690286 10.1016/j.canlet.2022.215780

[92] Bader J, Carson M, Enos R, Velazquez K, Sougiannis, Singh U, Becker W, Nagarkatti M, Fan D, Alexander, Murphy A. High-fat diet-fed ovariectomized mice are susceptible to accelerated subcutaneous tumor growth potentially through adipose tissue inflammation, local insulin-like growth factor release, and tumor associated macrophages. Oncotarget. 2020;11:4554–69. 33346251 10.18632/oncotarget.27832 PMC7733624

[93] Ma ZS, Li W. How and why men and women Differ in their microbiomes: Medical Ecology and Network analyses of the Microgenderome. Adv Sci (Weinh). 2019;6:1902054. 31832327 10.1002/advs.201902054 PMC6891928

[94] Ganci M, Suleyman E, Butt H, Ball M. Associations between self-reported psychological symptom severity and gut microbiota: further support for the microgenderome. BMC Psychiatry. 2022;22:307. 35501777 10.1186/s12888-022-03947-7 PMC9059404

[95] Song CH, Kim N, Nam RH, Choi SI, Jang JY, Choi J, Lee HN. Anti-PD-L1 antibody and/or 17beta-Estradiol treatment induces changes in the gut microbiome in MC38 Colon tumor model. Cancer Res Treat. 2023;55:894–909. 36634616 10.4143/crt.2022.1427 PMC10372611

[96] Song CH, Kim N, Nam RH, Choi SI, Lee HN, Surh YJ. 17beta-Estradiol supplementation changes gut microbiota diversity in intact and colorectal cancer-induced ICR male mice. Sci Rep. 2020;10:12283. 32704056 10.1038/s41598-020-69112-w PMC7378548

[97] Li Q, Chan H, Liu WX, Liu CA, Zhou Y, Huang D, Wang X, Li X, Xie C, Liu WY, et al. Carnobacterium maltaromaticum boosts intestinal vitamin D production to suppress colorectal cancer in female mice. Cancer Cell. 2023;41:1450–e14651458. 37478851 10.1016/j.ccell.2023.06.011

[98] Ibrahim A, Hugerth LW, Hases L, Saxena A, Seifert M, Thomas Q, Gustafsson JA, Engstrand L, Williams C. Colitis-induced colorectal cancer and intestinal epithelial estrogen receptor beta impact gut microbiota diversity. Int J Cancer. 2019;144:3086–98. 30515752 10.1002/ijc.32037 PMC6519213

[99] Leung HKM, Lo EKK, Chen C, Zhang F, Felicianna, Ismaiah MJ, El-Nezami H. Zearalenone attenuates colitis associated colorectal tumorigenesis through Ras/Raf/ERK pathway suppression and SCFA-producing bacteria promotion. Biomed Pharmacother. 2023;164:114973. 37269808 10.1016/j.biopha.2023.114973

[100] Yoon K, Kim N. Roles of sex hormones and gender in the gut microbiota. J Neurogastroenterol Motil. 2021;27:314–25. 33762473 10.5056/jnm20208 PMC8266488

[101] Santos-Marcos JA, Mora-Ortiz M, Tena-Sempere M, Lopez-Miranda J, Camargo A. Interaction between gut microbiota and sex hormones and their relation to sexual dimorphism in metabolic diseases. Biol Sex Differ. 2023;14:4. 36750874 10.1186/s13293-023-00490-2 PMC9903633

[102] Wu Z, Huang Y, Zhang R, Zheng C, You F, Wang M, Xiao C, Li X. Sex differences in colorectal cancer: with a focus on sex hormone-gut microbiome axis. Cell Commun Signal. 2024;22:167. 38454453 10.1186/s12964-024-01549-2 PMC10921775

[103] Baker JM, Al-Nakkash L, Herbst-Kralovetz MM. Estrogen-gut microbiome axis: physiological and clinical implications. Maturitas. 2017;103:45–53. 28778332 10.1016/j.maturitas.2017.06.025

[104] Parida S, Sharma D. The Microbiome-Estrogen connection and breast Cancer risk. Cells. 2019;8:1642. 31847455 10.3390/cells8121642 PMC6952974

[105] Kaliannan K, Robertson RC, Murphy K, Stanton C, Kang C, Wang B, Hao L, Bhan AK, Kang JX. Estrogen-mediated gut microbiome alterations influence sexual dimorphism in metabolic syndrome in mice. Microbiome. 2018;6:205. 30424806 10.1186/s40168-018-0587-0 PMC6234624

[106] Chakraborty B, Byemerwa J, Krebs T, Lim F, Chang CY, McDonnell DP. Estrogen Receptor Signaling in the Immune System. Endocr Rev. 2023;44:117–41. 35709009 10.1210/endrev/bnac017

[107] Hu S, Ding Q, Zhang W, Kang M, Ma J, Zhao L. Gut microbial beta-glucuronidase: a vital regulator in female estrogen metabolism. Gut Microbes. 2023;15:2236749. 37559394 10.1080/19490976.2023.2236749 PMC10416750

[108] Ervin SM, Li H, Lim L, Roberts LR, Liang X, Mani S, Redinbo MR. Gut microbial beta-glucuronidases reactivate estrogens as components of the estrobolome that reactivate estrogens. J Biol Chem. 2019;294:18586–99. 31636122 10.1074/jbc.RA119.010950 PMC6901331

[109] Ly LK, Doden HL, Ridlon JM. Gut feelings about bacterial steroid-17,20-desmolase. Mol Cell Endocrinol. 2021;525:111174. 33503463 10.1016/j.mce.2021.111174 PMC8886824

[110] Vom Steeg LG, Klein SL. Sex steroids mediate bidirectional interactions between hosts and microbes. Horm Behav. 2017;88:45–51. 27816626 10.1016/j.yhbeh.2016.10.016 PMC6530912

[111] Snigdha S, Ha K, Tsai P, Dinan TG, Bartos JD, Shahid M. Probiotics: potential novel therapeutics for microbiota-gut-brain axis dysfunction across gender and lifespan. Pharmacol Ther. 2022;231:107978. 34492236 10.1016/j.pharmthera.2021.107978

[112] Pal U, Ghosh S, Limaye AM. DNA methylation in the upstream CpG island of the GPER locus and its relationship with GPER expression in colon cancer cell lines. Mol Biol Rep. 2020;47:7547–55. 32936384 10.1007/s11033-020-05817-5

[113] Yuan X, Ruan W, Bobrow B, Carmeliet P, Eltzschig HK. Targeting hypoxia-inducible factors: therapeutic opportunities and challenges. Nat Rev Drug Discovery. 2023;23:175–200. 38123660 10.1038/s41573-023-00848-6 PMC12337356

[114] Xie M, Liang JL, Huang HD, Wang MJ, Zhang T, Yang XF. Low doses of Nonylphenol promote growth of Colon cancer cells through activation of ERK1/2 via G protein–coupled receptor 30. Cancer Res Treat. 2019;51:1620–31. 31096733 10.4143/crt.2018.340 PMC6790866

[115] Buhler M, Fahrlander J, Sauter A, Becker M, Wistorf E, Steinfath M, Stolz A. GPER1 links estrogens to centrosome amplification and chromosomal instability in human colon cells. Life Sci Alliance 2023, 6. 10.26508/lsa.202201499 PMC9670797 36384894

[116] Gilligan LC, Gondal A, Tang V, Hussain MT, Arvaniti A, Hewitt AM, Foster PA. Estrone Sulfate Transport and Steroid Sulfatase Activity in Colorectal Cancer: implications for hormone replacement therapy. Front Pharmacol. 2017;8:103. 28326039 10.3389/fphar.2017.00103 PMC5339229

[117] Santolla MF, Lappano R, De Marco P, Pupo M, Vivacqua A, Sisci D, Abonante S, Iacopetta D, Cappello AR, Dolce V, Maggiolini M. G protein-coupled estrogen receptor mediates the up-regulation of fatty acid synthase induced by 17beta-estradiol in cancer cells and cancer-associated fibroblasts. J Biol Chem. 2012;287:43234–45. 23135268 10.1074/jbc.M112.417303 PMC3527911

[118] Lo EKK, Lee JC, Turner PC, El-Nezami H. Low dose of zearalenone elevated colon cancer cell growth through G protein-coupled estrogenic receptor. Sci Rep. 2021;11:7403. 33795755 10.1038/s41598-021-86788-w PMC8016995

